# A State-of-Art on the Development of Nafion-Based Membrane for Performance Improvement in Direct Methanol Fuel Cells

**DOI:** 10.3390/membranes12050506

**Published:** 2022-05-10

**Authors:** Wei Wuen Ng, Hui San Thiam, Yean Ling Pang, Kok Chung Chong, Soon Onn Lai

**Affiliations:** 1 Department of Chemical Engineering, Lee Kong Chian Faculty of Engineering & Science, Sungai Long Campus, Universiti Tunku Abdul Rahman, Jalan Sungai Long, Bandar Sungai Long, Kajang 43000, Malaysia; wendy0727@1utar.my (W.W.N.); pangyl@utar.edu.my (Y.L.P.); chongkc@utar.edu.my (K.C.C.); laiso@utar.edu.my (S.O.L.); 2 Centre for Photonics and Advanced Materials Research, Universiti Tunku Abdul Rahman, Kajang 43000, Malaysia

**Keywords:** direct methanol fuel cell, Nafion, PEM, proton conductivity, methanol permeability

## Abstract

Nafion, a perfluorosulfonic acid proton exchange membrane (PEM), has been widely used in direct methanol fuel cells (DMFCs) to serve as a proton carrier, methanol barrier, and separator for the anode and cathode. A significant drawback of Nafion in DMFC applications is the high anode-to-cathode methanol fuel permeability that results in over 40% fuel waste. Therefore, the development of a new membrane with lower permeability while retaining the high proton conductivity and other inherent properties of Nafion is greatly desired. In light of these considerations, this paper discusses the research findings on developing Nafion-based membranes for DMFC. Several aspects of the DMFC membrane are also presented, including functional requirements, transport mechanisms, and preparation strategies. More importantly, the effect of the various modification approaches on the performance of the Nafion membrane is highlighted. These include the incorporation of inorganic fillers, carbon nanomaterials, ionic liquids, polymers, or other techniques. The feasibility of these membranes for DMFC applications is discussed critically in terms of transport phenomena-related characteristics such as proton conductivity and methanol permeability. Moreover, the current challenges and future prospects of Nafion-based membranes for DMFC are presented. This paper will serve as a resource for the DMFC research community, with the goal of improving the cost-effectiveness and performance of DMFC membranes.

## 1. Introduction

Immense consumption of unsustainable and non-renewable fossil fuels for a variety of purposes, including transportation, generation, and conversion, has greatly reduced the availability of existing energy resources such as petroleum, coal, or natural gas [1]. The usage of fossil fuels also contributes to environmental degradation due to the high amount of greenhouse gas emissions. As energy demands rise and environmental concerns grow, it is imperative that long-term renewable energy sources be developed. However, renewable sources such as solar, wind, or geothermal are limited by the weather conditions. Thereby, in the present juncture of energy and economic insecurity, the fuel cell has emerged as a potential alternative to conventional energy sources due to the numerous advantages it offers. Particularly, fuel cell establishes high energy density, lower emission of pollutants such as SO_X_, NO_X_, CO, and CO_2_, and has the benefit of being portable.

The fuel cell is an electrochemical device that supplies energy continuously in one step by converting chemical energy into electrical energy as long as the external supply of fuel and oxidant is maintained [2]. Thus, fuel cells are able to provide power continuously without requiring long charging times or the replacement of new energy generators. Presently, there are several types of fuel cells, such as alkaline fuel cells (AFC), phosphoric acid fuel cells (PAFC), molten carbonate fuel cells (MCFC), solid oxide fuel cells (SOFC), and proton exchange membrane fuel cells (PEMFC), which are classified based on the type of electrolytes used and operating conditions [3]. Electrolytes such as alkaline solution, acidic solution, molten carbonate salt, ceramic ion, and solid polymer are used in AFC, PAFC, MCFC, SOFC, and PEMFC, respectively. Moreover, fuel cells differ by the reactants used, the type of ions transported, the structure of the fuel cell system, and their application in different fields. Among all these fuel cells, PEMFC has been actively researched owing to its low operating temperature of less than 100 °C, high power density, low corrosion, facile infrastructure (no moving parts), and quiet operation [4]. Generally, PEMFC can operate on a variety of fuel sources, for instance, hydrogen, formic acid, or alcohol.

The direct methanol fuel cell (DMFC) is one of the PEMFCs, which uses methanol directly as fuel to generate electricity without fuel combustion. DMFC has gained much attention as it is a simple and compact system that eliminates the need for auxiliary units, as well as has a fast start-up time under ambient conditions [5,6]. Moreover, liquid methanol is low cost, environmentally friendly, easy to distribute, and stores and handles under standard conditions. Methanol also has a high energy density [7,8]. According to Joghee et al., the energy density of methanol is 15 times higher than lithiumion batteries [9]. As a result, it benefits portable devices, especially when conventional batteries are unable to meet growing energy demands, as people nowadays expect more functions that require a continual supply of power. In addition, methanol is intriguing as it can be produced from biomass, which is considered a cleaner energy source for long-term usage.

The basic configuration of a single DMFC consists of a fuel reservoir, bipolar current collectors, and a membrane electrode assembly (MEA). MEA is made up of a solid polymer electrolyte membrane (PEM), which is the core component of DMFC, sandwiched between two catalyzed electrodes. The electrode comprises two layers, namely the catalyst layer and the gas diffusion layer (GDL). Carbon-supported PtRu and carbon-supported Pt catalyst are widely used in anode and cathode to speed up the methanol oxidation reaction (MOR) and oxygen reduction reactions (ORR), respectively [7]. However, due to the scarcity and high cost of Pt, much effort has been expended on producing low-cost and efficient dual-role electrocatalysts for MOR and ORR [10]. On the other hand, GDL, which is placed in intimate contact with the catalyst layer, works to conduct electrons from the catalyst layer to the current collector, as well as a supportive or protective layer for the catalyst by offering suitable mechanical strength [11]. Typically, GDL is made to establish necessary hydrophobic characteristics for carbon dioxide to escape from the anode and to retain water within PEM, preventing it from drying up [9].

PEM is an ion-exchange membrane with a fixed charge; it is responsible for transporting oppositely charged ions from anode to cathode during electrochemical reactions. With that said, a cation exchange membrane is composed of a negatively charged group with a free proton ion and serves as a cation carrier and a barrier to anion [12]. Thus, in DMFC, PEM is also known as the proton exchange membrane, as it is used to transport protons from anode to cathode. Apart from its function as a proton transporter, the PEM provides a barrier for methanol fuel and electrons, enabling current to flow in the external circuit. As a result, PEM has crucial effects on the efficiency and performance of DMFC.

Nafion, also known as perfluorinated sulfonic acid (PFSA) membrane, was developed by DuPont and is currently the most widely used and accepted PEM. It consists of a hydrophobic polytetrafluoroethylene (PTFE) backbone and a hydrophilic perfluorinated vinyl ether pendant side chain that is ionically bonded to a sulfonic acid group (−SO_3_H) [13]. The negatively charged group (SO_3_^−^) of Nafion will block anions but enable cations to pass through. This ionic hydrophilic group also takes up water to keep the membrane hydrated, which assists in proton migration. When water is absorbed by the sulfonic acid groups, the hydrophilic ion-cluster domains and water bridges are formed to act as proton migration channels, enhancing the proton transfer [14]. The proton conductivity of the Nafion membrane can approach 0.1 s/cm when fully hydrated and at room temperature [15]. As for the PTFE backbone, it consists of high electronegativity small-sized fluoride atoms connected by a strong C-F bond, which contributes to the suitable mechanical properties of Nafion in a water-swollen state and its chemical stability [1,16]. As a result, Nafion may function in a fuel cell for more than 60,000 h, providing an outstanding lifespan for Nafion [17].

Given the vital significance of PEM in DMFC, discovering and developing suitable PEMs is a critical aspect of DMFC commercialization. To date, numerous review articles [1,13,18,19] have been published that outline the various types of membranes developed for use in PEMFCs. However, discussions of developing PEM, particularly for DMFC, have not been widely published thus far. With the increased attention and tremendous advancement that DMFC has seen in the last several years, a comprehensive and up-to-date review of the Nafion membrane, which is the most often used in DMFC, is important. Therefore, this paper provides a detailed overview of the development of Nafion-based PEMs designed exclusively for use in DMFCs.

## 2. Functional Requirements

DMFC has been extensively examined and tested with various membranes. These membranes were designed to fulfill multiple functions at the same time. Hence, this section focuses on the functional needs of membranes that can be used in DMFCs.

### 2.1. High Proton Conductivity

A membrane with excellent proton conductivity can prevent ohmic loss and promote mass transport of protons, which is advantageous for supporting high current densities. For DMFC operation, the proton generated from the methanol oxidation at the anode should be able to migrate effectively through the membrane. The proton-conducting functionalities such as sulfonic acid (−SO_3_H), phosphonic acid (–PO_3_H), carboxylic acid (–COOH), or amine (–NH_2_) within the polymer chains are responsible for the formation of ionic clusters for proton migration by forming bonds with the hydronium ion, to which the proton is provisionally attached [20]. Therefore, a high density of proton-conducting groups (–SO_3_^−^, –PO_3_^−^, –COO^−^, or –NH_2_) will facilitate proton transportation. Solid acids such as heteropolyacids (HPA), including phosphotungstic acid (PWA) and silicotungstic acid (SiWA), zirconium phosphate (ZrP), or cesium salts of HPA, have been reported to enhance proton conductivity by increasing the concentration of acid functional groups [19]. External factors, for instance, operating temperature and relative humidity (RH), may also have a plausible effect on proton conductivity. The conductivity of the proton was shown to increase with temperature [21]. Water content in the membrane also contributes to proton conductivity, as water forms swollen and connected ionic clusters that allow proton hopping and diffusion. However, the humidity of PEM is temperature-dependent. Under high-temperature operating conditions, the membrane may dehydrate. Thus, numerous approaches have been explored to improve the water retention capacity of membranes for use at high temperatures, including the addition of amphiphilic and hydrophilic fillers to membranes, such as silica and poly (vinyl alcohol) [22,23]. Additionally, the structure of PEM, such as ionic cluster size, the density of acidic functionalities, the tortuosity, as well as the interaction between the filler and polymer, all will influence the proton conductivity.

### 2.2. Low Methanol Permeability

Another important property of a membrane suitable for use in DMFCs is its suitable resistance to methanol. Methanol diffuses concurrently with protons by means of a bulk transport mechanism that uses free water molecules as transport agents inside the polymer matrix. According to Ahmad et al., the crossing of methanol from anode to cathode in Nafion wastes over 40% of the methanol fuel in DMFC. Methanol crossover also has a detrimental effect on the performance and durability of DMFC [24]. It leads to a reduction in fuel efficiency and open-circuit voltage, as well as poisoning the electrode due to direct oxidation of methanol at the cathode [6,25]. Therefore, extremely low methanol permeability is necessary to maximize fuel consumption and coulombic efficiency. According to reports, methanol permeability increases with temperature and is concentration-dependent [26]. The permeability of methanol also depends on the current density. Methanol crossover rate decreases as current density increases, as more methanol is consumed at high current density, reducing the concentration gradient in the system. Additionally, the alignment, orientation, and local packing density of the membrane matrix all affect methanol permeability, as reported by [27].

### 2.3. High Electrical Resistivity

PEMs for DMFCs should be capable of rejecting electron transport and driving them to an external circuit for electricity generation. For every proton that is transferred via the electrolyte, an equal amount of electrons must be transported through the external circuit, where they combine at the cathode to form water.

### 2.4. Suitable Chemical Stability

Chemical stability is also important for a PEM in DMFCs to be widely used and commercialized. The chemical durability of the membrane is a factor that affects the lifetime of fuel cells. In the operation of a fuel cell, the membrane will be subjected to a chemically oxidizing environment on the anode side and a chemically reducing environment on the cathode side. At the cathode, hydrogen peroxide is formed when oxygen is reduced through the two-electron pathway [28]. When hydrogen peroxide decomposes and reacts with metal ions (e.g., Fe^2+^, Cu^2+^, and Cr^3+^) formed during the degradation of other components in the fuel cell (e.g., bipolar plate and sealing materials), intermediate products, such as hydroxyl radicals with strong oxidative characteristics are produced [29]. These radicals attack the polymer chain, causing defragmentation, unzipping, and thinning of the membrane [30,31]. Therefore, a stable membrane is required in DMFC to resist the chemical degradation caused by free radicals. The indirect approach to minimizing the effect of reactive radicals is to improve the membrane stability through the synthesis of short side-chain polymers or modification of hydrocarbon polymer electrolytes. While some publications also reported direct methods for mitigating free radical degradation, such as preventing the formation of hydrogen peroxide, destroying hydrogen peroxide, or incorporating free radical scavengers to suppress their reactivity and capture reactive oxygen radicals before they attack the membrane [16,30]. Cerium oxide, a metal oxide with valence electrons, is one of the materials used as a free radical scavenger in PEM.

### 2.5. Suitable Mechanical Stability

Mechanical strength is critical as it is closely related to the methanol crossover phenomenon, which affects DMFC performance. During fuel cell operation, the membrane undergoes dimensional changes due to the repeated swelling and shrinking processes [32]. The non-uniformly stress and compression exerted on the membrane lead to the formation of cracks and pinholes that have the potential to propagate widely across the membrane. Additionally, the presence of radicals arises from the decomposition reaction of hydrogen peroxide, resulting in the formation of local defects that speeds up mechanical damage to the membrane [30]. All these defects will further worsen methanol crossover issues. Therefore, a membrane with high flexibility and low rigidity is preferred to sustain mechanical stresses and prevent the membrane from breaking or perforating. Carbon nanomaterials are a type of filler material that has been used as reinforcing agents in polymer membranes due to their high mechanical stability. A packed structure is created within the polymer matrix, allowing it to withstand fatigue stress [33].

All of the above functional requirements have a big impact on the performance of the membrane and the whole DMFC system. Despite substantial research into alternative membrane materials that meet the requirements, Nafion remains the most widely used commercial membrane in DMFC. As such, this review paper focuses on modifications to the Nafion membrane that improve its proton conductivity, methanol-blocking capability, and mechanical and chemical stability.

## 3. Proton Transport Mechanism in Nafion

Proton transportation across PEM is mainly carried out through surface diffusion, Grotthuss mechanism (hopping), or vehicular mechanism (diffusion) [34]. At the interface of a water-filled channel or pore wall, proton transfer occurs via surface diffusion, in which the proton hops between the adjacent sulfonic acid groups. In the context of the Grotthuss mechanism, protons jump in the percolation network formed by water molecules within the swollen hydrated ionic cluster [35]. Protons attached to sulfonic acid groups will provisionally bind to water molecules in the hydronium (H_3_O^+^) form. The protons are then transferred by breaking the hydrogen bond with one water molecule and forming a new hydrogen bond with another water molecule nearby [16,34]. Each water molecule works simultaneously to bond a free proton and release another in this process. Thus, increasing the water content within PEM facilitates Grotthuss proton transportation since protons can be transported faster to a closer water molecule [12]. On the other hand, vehicular diffusion, in which proton migrates together with a water molecule in the form of hydronium ion (H_3_O^+^), Zundel (H_5_O_2_^+^), or Eigen (H_9_O_4_^+^) is driven by the concentration gradient and electroosmosis drag [16,36]. In other words, protons diffuse across the membrane, with water molecules acting as the “vehicle”. Apart from that, the free volume within the polymer chains is also essential to the functioning of the vehicular mechanism.

Proton transportation, according to both Grotthuss and vehicular mechanisms, is highly dependent on the level of hydration, as water molecules participate in proton transport. However, membranes lose water at temperatures lower than 0 °C or higher than 100 °C [35]. At low degrees of hydration, the diffusion of water molecules is retarded, and connectivity between water molecules becomes poor, resulting in a low interaction of protons with the immobile sulfonic acid group [19,37]. Surface diffusion, in turn, dominates when there is insufficient water, as protons can only be transported by forming hydrogen bonds with sulfonic acid groups [11]. All in all, the proton conductivity depends on all three transport mechanisms, which are affected by the operating temperature, water content, and the inherent properties of the membrane.

## 4. Preparation Methods

Apart from the operating conditions, the membrane preparation method has a direct impact on the properties of the membrane and hence its performance in DMFCs. To date, there are various methods for synthesizing Nafion-based membranes; the three commonly used are sol-gel, blending, and multilayer membrane technology.

### 4.1. Blending

Solution or melt blending is the simplest and most efficient way of preparing polymer membranes or films [38,39]. In general, this synthesis method uses pre-made additives such as inorganic particles or polymers that are mixed directly in the Nafion solution. Melt blending is a term that refers to the process by which components are melted under high temperatures prior to being mixed to form a miscible blend. The advancement of conventional blending has made it possible to incorporate the majority of fillers into the polymer matrix. After that, the solution-casting method can be used to make the blend membrane. The main shortcoming of this conventional blending approach is that the fillers are difficult to disperse well in the polymer matrix to obtain a homogeneous system [40]. In this regard, a solvent such as isopropyl acid is used in conjunction with the process. The solvents will be removed sequentially by heat treatment. In addition to the need for suitable dispersion, smaller fillers with a large surface area are preferred as they promote greater interaction between the polymer and the filler. On the other side, a higher content of filler in the blend prevents membrane fabrication due to phase separation and subsequent mechanical integrity loss [41]. Additionally, a high filler content results in the aggregation of fillers. To address this issue, surface functionalization was reported to provide uniform morphology of the Nafion composite membrane.

### 4.2. In Situ Sol-Gel

The sol-gel process is usually employed to modify Nafion with hygroscopic metal oxides, such as silica (SiO_2_), titania (TiO_2_), zirconia (ZrO_2_), and aluminum oxide (Al_2_O_3_), by establishing dynamic crosslinks with sulfonic acid groups in Nafion [42]. Sol refers to a colloidal suspension containing metal alkoxides as the precursor, which are only dissolved in alcohol. Water is added to the sol to initiate a sol-gel reaction between the metallic compound and water through hydrolysis and condensation, resulting in the growth of the network or formation of nanoparticles [43,44]. The sol-gel generated inorganic metal oxide nanoparticles can be introduced into Nafion in situ or ex situ. Conventional top-down ex situ methods use pre-synthesized nanoparticles that are subsequently blended or intercalated into a polymer. For in situ sol-gel synthesis, nanoparticles can be formed either in the presence of a pre-formed polymer or by forming both the organic and inorganic networks concurrently in a solution, resulting in an interpenetrating polymer network. The inorganic network in composites is formed through hydrolysis and condensation reactions, whereas the organic network is formed through polymerizations; combining the two networks leads to gel formation of Nafion/inorganic composite membrane. In the case of pre-formed polymers, such as Nafion, the host material serves as a template that directs the morphology, particle size, and growth rate of the oxide within the Nafion matrix, resulting in nanosized particles. In general, this approach involves immersing the Nafion membrane in water to swell the pore and allow for maximum precursor solution absorption. The membrane is then immersed in metal alkoxides solvated in alcohol and dried. Using this method, the particles are able to disperse homogeneously and form a completely transparent membrane as compared to the membranes produced by the blending method, which is cloudy. In addition, the in situ formation of inorganic particles is promising since the particle size and dispersion can be controlled by altering the concentration of the precursors. Thus, this strategy effectively eliminates the interface compatibility and cavity issues that exist between the filler and the polymer matrix in the chemical blend (ex situ mixing method) [45]. However, the particles formed by this procedure were evidently not as amenable to modification as those derived from ex situ sources. Ex situ sol-gel, in turn, faces the issue of nanoparticle aggregation, which results in inhomogeneity in the composite membrane and degrades its properties. The sol-gel method is also limited to inorganic metal oxides. It was found to be difficult to prepare membranes modified with other materials, such as carbon materials, using this approach.

### 4.3. Multilayer Method

The solubility of filler in the Nafion solution is the main concern while preparing a Nafion-based membrane since it might cause a decrease in proton conductivity. This impact can be mitigated by using a multilayer membrane produced via hot press or dip coating. This approach retains the properties of the material at each layer and does not disrupt the structure of the material. The term “hot press” refers to the process of pressing two or more separate membrane layers together under high temperatures. While dip coating can produce the thinnest membranes by dipping an initial membrane into a solution containing polymer or other modifiers. Thereby, the initial membrane will be attached to the solution on both sides. The dipping process can be repeated several times to obtain the desired thickness and characteristics [12].

Another technique for forming multilayer membranes is layer-by-layer (LbL) self-assembly, which is based upon alternate depositions or dipping of oppositely charged polyelectrolytes (polycation or polyanion) onto a membrane substrate. Typically, the surface of the Nafion membrane is coated with an alternating layer of the cationic or anionic polymer as a thin film. After each dipping cycle, the surface charge is reversed, which allows the deposition of the next layer. Electrostatic attraction between the cation and anion initiates ionic crosslinking, resulting in the formation of a nanometer-scale thin film with a controlled structure and composition that does not affect the mechanical and chemical stability of the substrate. A wide variety of materials could be deposited by the LbL method, including polyions, metals, ceramics, nanoparticles, and biological molecules [46]. LbL assembly successfully diminished methanol permeation across the membrane. However, LbL deters proton conductivity because most of the sulfonic acid groups in Nafion are engaged in ionic crosslinking, leaving only a few free to transport protons. This shortcoming can be minimized by increasing the content of free sulfonic acid groups and incorporating a suitable crosslinking density in the membrane. In this manner, unbalanced charges (SO_3_^−^) of polyampholyte (e.g., sulfonated cardo poly (arylene ether sulfone) (SPES-NH_2_)) and an effective crosslinker (e.g., glutaraldehyde) appear to be the strategy for achieving these objectives [47]. The amino group (-NH_2_) in SPES-NH_2_ formed a covalent bond with the aldehyde group in glutaraldehyde, leaving high content of free SO_3_H for proton transport.

Considering the different fabrication methods outlined before, it is worth mentioning that these are all viable approaches for synthesizing Nafion-based membrane for DMFC applications. However, each method has certain limitations that may alter the qualities of the membrane. Hence, further research and optimization are needed for different materials used to obtain an optimum DMFC membrane.

## 5. Modification Strategies

Numerous approaches have been developed and implemented in order to improve the performance of the Nafion membrane in DMFC. Some of these methods include blending with other polymers and adding inorganic components, ionic liquids, and carbon nanomaterials. This section discusses various studies that have been conducted using these approaches and found to significantly improve proton conductivity and reduce methanol permeability in DMFCs.

### 5.1. Polymeric Blend and Composite Membranes

Combining a variety of polymer materials with Nafion is a simple approach to enhance the selectivity of PEM. To reduce methanol crossover, synthetic polymers with low methanol compatibility, such as poly (vinyl alcohol) (PVA), polybenzimidazole (PBI), polyvinylidene fluoride (PVDF), and poly (aniline) (PANI) have been composited with Nafion [48]. Blending Nafion ionomer with miscible polymers also strengthens the polymeric membrane by lifting its mechanical stability [49]. However, the polymers exhibiting poorer proton conductivity due to the absence of charge functional groups, such as sulfonic acid (−SO_3_H) and carboxylic acid (-COOH) groups, are a bottleneck for their application in Nafion. Thus, natural or synthetic polymers are functionalized with high conductivity acidic groups such as sulfonic acid through chemical modification or grafting prior to being blended with Nafion to increase their conductivity [19].

**Figure 1:**
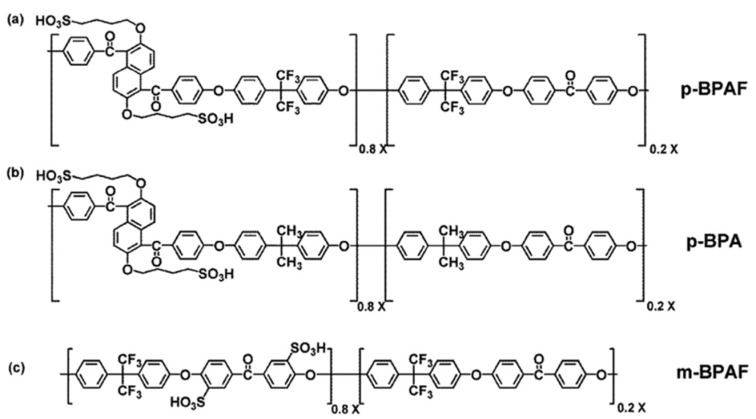
Chemical structures of three types of SPAEK blending modifiers: (**a**) p−BPAF; (**b**) p−BPA; (**c**) m−BPAF. Reprinted with permission from Ref. [48]. Copyright 2018 Elsevier.

Ru et al. blended sulfonated poly (arylene ether ketone) (SPAEK) with Nafion, and three different SPAEK@Nafion membranes with different structures were synthesized [48]. Sulfonic acid groups were grafted onto the main and side chains of SPAEK using chlorine treatment (denoted as m-BPAF and p-BPAF, respectively), as well as onto side chains of SPAEK without fluorine treatment (denoted as p-BPA). The SPAEK blending modifiers, namely p-BPAF, p-BPA, and m-BPAF, are represented in Figure 1a–c respectively. All sulfonated composite membranes performed better than pristine Nafion in terms of proton conductivity, yet composite membranes with sulfonic acid groups located at the pendant side chain enhanced better chain mobility and contributed to accelerated proton transport. To further improve the performance of SPAEK@Nafion composite membranes, the same authors [39] introduced a crosslinking agent, fluorinated epoxy resin monomer (fEO), into the composite membrane to form a semi-interpenetrating polymer network (semi-IPN). The resulted composite membrane (denoted as S@N/fEO) showed lower methanol permeability than pristine SPAEK@Nafion and Nafion 212 because of the crosslinking semi-IPN structures that constrain the mobility of molecular segments and hence prevent methanol passage. Additionally, the SPAEK blender, which comprises an alkylsulfonic acid group on the side chain and fluorine moieties on the main chain, can alter the microstructure, resulting in a reduced distance between ionic clusters and facilitating proton conduction. As a result, S@N/fEO demonstrated an almost 1.3-fold increase in selectivity and DMFC performance over Nafion 212. In another attempt, Li et al. reported the use of sulfonated 1,5-Bis(4-fluorobenzoyl)-2,6-dimethoxynaphthalene and 4,4-difluorobenzophenone (SDF-PAEK) as the skeleton molecule of the Nafion membrane to induce the automatic formation of hydrophilic-hydrophobic phase separation [21]. SDF-PAEK was composed of a hydrophobic phenyl and naphthyl ring backbone as well as trifluoromethyl side chains with sulfonic acid groups at the end of the side chain. With 15 wt% of skeleton molecules, the SDF-PAEK@Nafion composite membrane showed the highest proton conductivity of 0.197 S/cm and half the methanol permeability (2.03 × 10^−6^ cm^2^/s) of the recast Nafion (4.08 × 10^−6^ cm^2^/s). Thus, the selectivity of SDF-PAEK@Nafion-15% (9.73 × 10^4^ S s/cm^3^) was more than 2.6 times that of recast Nafion (3.71 × 10^4^ S s/cm^3^), and a maximum power density of 139 mW/cm^2^ was achieved at 80 °C. The improvements also reduced the Nafion composition by 20%, resulting in a significant cost reduction.

Apart from SPAEK, a combination of PAni and Nafion has been researched for developing a practical and efficient PEM. For example, Gonzalez-Ausejo et al. modified Nafion with low-cost PAni through two different in situ polymerization protocols, grafting PAni onto the Nafion surface (contact method) and blending PAni into the polymer matrix (crossover route) [50]. The proton conductivity and water uptake of both composite membranes decreased remarkably with increased PAni particle incorporation, signifying that PAni acts as an obstructive hindrance toward the flow channel. Despite the unfavorable conductivity results, both grafting and blending PAni with Nafion resulted in a significant reduction in methanol permeability. This is because the PAni is shown not only on the surface but also within the ionic cluster, where polymerization took place and reduced the free volume of the percolation channel [51]. With regards to the crucial role of PAni as a methanol barrier, Escudero-Cid et al. synthesized Nafion/PAni through in situ electrochemical and chemical polymerization [52]. Nafion/PAni membranes produced in either way possessed two times lower proton conductivity than Nafion 117. The decrement agrees with the previous result in which a strong hydrogen bond between the amine groups in PAni and the sulfonic groups in Nafion, as well as the behavior of PAni as a pore-filling agent, obstruct the conducting pathway. This compact structure, in turn, led to a radical decrease in methanol permeability. The power density for the PAni-modified Nafion membrane was found to be seven times higher than that of unmodified Nafion and a two-fold increase in durability. Another workable concept for tuning Nafion/PAni membrane is to prepare partially sulfonated PAni (SPAni) by sulfonating it with chlorosulfonic acid and blending it with Nafion, as reported by Dutta, Das, and Kundu [41]. As proven by the increment in ion exchange capacity (IEC) value, SPAni implanted extra capacities for proton uptake through the sulfonic acid groups. The increased proton conductivity is also due to the lone pair electrons on the nitrogen atom, which interacts with protons in acidic conditions. Furthermore, pore-blocking with increasing content of SPAni to 30 wt% depressed methanol diffusion by a factor of more than one order of magnitude. However, the effect of SPAni on blocking the conduction channel surpassed its advantageous structure for proton migration, which reduced the proton conductivity of the composite membrane compared to pristine Nafion. The authors then suggested that a higher degree of sulfonation on SPAni would be possible to overcome the discrepant behavior of SPAni.

**Figure 2:**
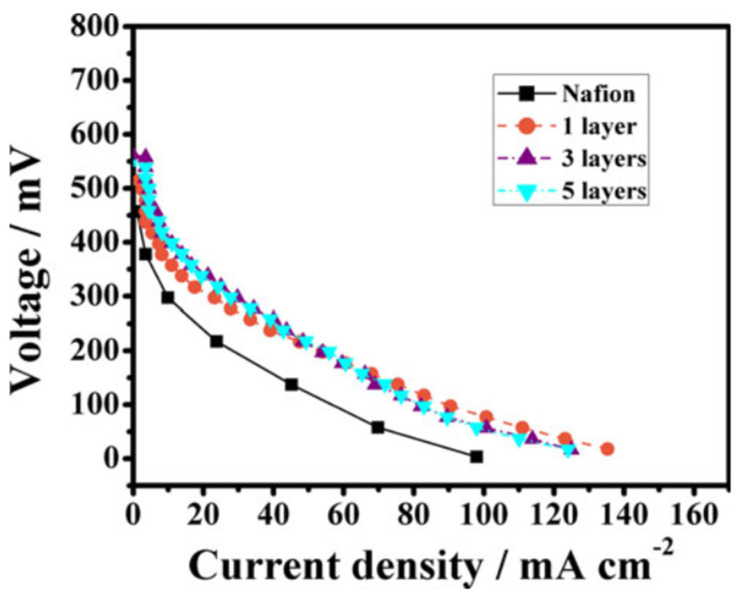
MEA performance of Nafion impregnated multilayer PVDF fibrous membranes with 10 M methanol. Reprinted with permission from Ref. [54]. Copyright 2019 Wiley-VCH.

Another effective effort to improve the methanol resistance of Nafion membrane was made by Cho, Park, and Jung, who blended polyvinylidene difluoride (PVdF) copolymer with Nafion in the presence of DMF or acetone as solvent [53]. The methanol permeability of the mixed membrane was found to be greatly reduced, albeit at the expense of proton conductivity, which resulted in a slightly lower power density output than Nafion. To further optimize the use of PVdF in Nafion, Li et al. impregnated thin layer(s) of electrospun PVdF nanofiber between layers of Nafion membranes [54]. When tested under 10 M methanol, it was found that as the number of PVdF fiber mats increased, the Nafion/PVdF membranes exhibited significantly decreased methanol permeability and swelling ratio at the expense of a slight reduction in proton conductivity. As a result, as shown in Figure 2, the composite membranes showed better performance, with a current density of 55 mA/cm^2^ exceeding that of Nafion (28 mA/cm^2^). Additionally, the thermal stability of Nafion/PVdF was improved from 500 °C to 700 °C before complete degradation.

PVdF can be mixed with hexafluoropropilene (HFP) to form a copolymer known as PVdF-co-HFP polymer and blended with Nafion. Kumar et al. investigated the crosslinked sulfonated polystyrene (SPS) in the blend of poly (vinylidene fluoride)-co-hexafluoropropylene/Nafion (PVdF-co-HFP/Nafion) in a bid to promote water uptake and proton conductivity [55]. PVdF-co-HFP/Nafion exhibited compelling chemical stability and methanol resistivity but lacked enough hydrophilicity. In view of this issue, the greater polarity of SPS, which is capable of absorbing water, could be seen to improve water uptake considerably by up to 24%. Compared to the proton conductivity of Nafion 117 of 3.02 × 10^−2^ s/cm, the proton conductivity of the composite membrane containing 20 wt% SPS was increased to 3.16 × 10^−2^ s/cm due to the enhanced water uptake. The methanol permeability of Nafion 117 and composite membranes was found to be comparable, owing to the free volume within the membrane allowing for swelling and passage of methanol. Despite this, because of the low Nafion content of the blend, the cost of the PEM is reduced. Kumar and Kundu proceeded to exploit sulfonated PVdF-co-HFP as a coating for Nafion 117 [56]. The laminated membrane effectively blocked methanol molecules by two orders of magnitude higher than plain Nafion, resulting in higher selectivity and power density to allow for consumption of higher methanol concentration at the anode. Mondal, Soam, and Kundu conducted another study in which a blend of sulfonated PVdF-co-HFP and PBI layer was laminated on Nafion to improve the methanol barrier properties without sacrificing the proton conductivity [57]. The proton conductivity of the composite membrane increased inevitably as a result of the additional free sulfonic acid groups, which boosted water uptake and formed connected hydrophilic channels and hopping sites that facilitated proton migration. Methanol permeation was reduced as a result of the formation of an acid-base complex between Nafion and PBI, in which PBI diffuses into the Nafion matrix and blocks the pores of the Nafion membrane. Additionally, the paper highlights the improvement in the thermal and mechanical stability of the laminated membrane. It is worthwhile to note that unanimous consensus was achieved on the efficacy of functionalized polymer in improving DMFC performance.

Hydrophilic PVA, which is frequently used to separate alcohol and water in the pervaporation process, has also been considered a suitable choice for modifying Nafion. PVA has a higher water affinity relative to methanol [58], and it is a biodegradable semi-crystalline synthetic polymer with excellent thermal and chemical stability [59]. The abundance of hydroxyl groups present in PVA enables it to create hydrogen bonds with sulfonic acid groups of Nafion, allowing chemical changes in the polymer chain to take place, resulting in excellent film formation. Due to the strong hydrogen interaction within the composite matrix that restricts the methanol transport channel, a membrane with a lower methanol permeability can be obtained. Lin et al. investigated this prospective material by synthesizing Nafion/PVA nanofiber composite and Nafion/PVA blend membranes for DMFC [60]. Nafion/PVA nanofiber and Nafion/PVA blend containing 10 wt% and 5 wt% of PVA, respectively, outperformed Nafion 117 and Nafion 112. Similarly, Romano et al. investigated the feasibility of using PVA as a modifier in Nafion [23]. Despite the protonic resistance, the Nafion/PVA composite membrane displayed equivalent performance to pristine Nafion at 95 °C and 2 M methanol concentration, owing to its high methanol-blocking capacity. In a more recent study by Zizhou et al., PVA/Nafion nanofibrous membranes were synthesized through electrospinning, followed by either thermal treatment or chemical crosslinking with BTSA, and finally sulfonation [61]. The crosslinked sulfonated PVA/Nafion showed comparable thermal stability (up to 200 °C) with Nafion, while the thermally treated PVA/Nafion was found to have higher oxidative stability but a lower IEC value than Nafion due to the crystallization induced by the hydrogen bond between the hydroxyl group of PVA and sulfonic groups of Nafion.

Rather than using the conventional blending and coating methods, polymer composites can be created by grafting Nafion membranes with a variety of functionalized polymers and initiation systems. Mohy Eldin et al. [62] modified a commercial Nafion 117 membrane by grafting poly (glycidyl methacrylate) (PGMA) onto it using a persulphate initiation system, followed by sulfonation of the epoxy groups in PGMA. The grafted PGMA alters the structure of the Nafion membrane and decreases the channel size by occupying the amorphous free volume in the membrane. As a result, the methanol crossover was reduced to 45.36% of its value for the virgin Nafion. Sulfonation of the grafted membrane also secured the IEC of the membrane, hence maintaining its ionic conductivity. Thus, the performance factor (IEC/methanol permeability) of the modified membrane (584 × 10^−9^) was higher than the virgin Nafion membrane (306 × 10^−9^). Another approach to grafting functionalized polymers, sulfonated polystyrene, on a commercial Nafion-115 membrane was reported by Arslanova et al. [63]. Nafion-115 was modified with crosslinked polystyrene prepared via radical polymerization under argon purge, and the membrane was then sulfonated. By constructing rigid transport channels, this approach was found to prevent the collapse of channels necessary for effective proton transport upon drying. Additionally, the sulfonated polystyrene participates in ionic transport, resulting in a 33–34% improvement in proton conductivity at lower relative humidity (32%) compared to the original Nafion membrane. These findings suggest that using grafting polymerization to enhance the performance of commercial Nafion membranes is a promising strategy.

Although numerous approaches are being taken to develop PEM with low methanol permeation and suitable proton conductivity, there are few studies on how to modify PEM to achieve sustainable and durable performance. Recently, a somewhat different and interesting idea of extending the lifespan of Nafion membranes by making them self-heal and restoring their intrinsic properties was proposed by Li and co-workers [64]. The authors shed new light on the self-healable Nafion-based PEM derived from PVA and 4-carboxybenzaldehyde (CBA). The CBA-modified Nafion-PVA membrane could repair the mechanical damage through the formation of reversible dynamic hydrogen bonding between Nafion and PVA. Thus, the membrane could self-heal intrinsically, restore the proton conductivity and methanol resistance by rebuilding the hydrogen bonds and covering up the defects. On the other hand, CBA consists of benzoic acid groups that can form dense proton-conducting pathways, which results in a proton conductivity 1.5 times higher than an unmodified Nafion membrane.

**Figure 3:**
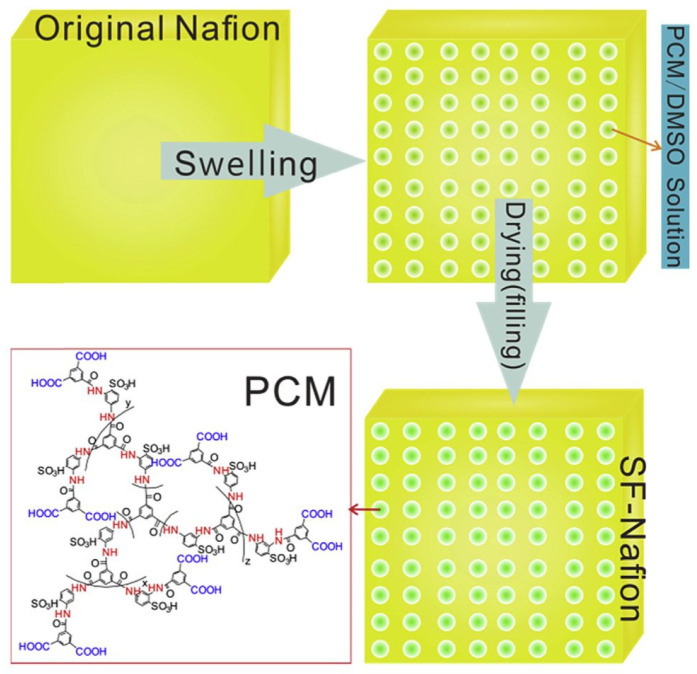
Schematic illustration of in situ swelling−filling Nafion modification strategy. Reprinted with permission from Ref. [65]. Copyright 2016 Elsevier.

Other than the aforementioned materials, polyamide, polyacrylonitrile (PAN), polypyrrole, and cellulose nanocrystal were also used to prepare Nafion blends polymer PEM. One of these studies investigated the adoption of an in situ nanoscale swelling-filling (SF) protocol to fill the cavities within Nafion with a proton-conductive molecule (PCM) (Figure 3), namely hyperbranched polyamide [65]. It is worth mentioning that the Nafion chain was left intact by applying the SF approach by using dimethyl sulfoxide (DMSO) as a swelling solvent. The strong electrostatic interaction between the dense structure of the PCM and Nafion increased the mechanical strength substantially. The PCM-filled Nafion was capable of physically blocking methanol molecules, contributing to a 17% reduction in methanol permeability while increasing proton conductivity by about 45%. As a result, the power density of DMFC with the SF-treated membrane was uplifted by 33%. To get rid of the formation of micro-pores in the SF-treated membrane, Xu et al. modified the structure of the PCM filler to generate nano-shuttle (NH) PCM [66]. The NH-Nafion membrane showcased excellent proton conductivity, with a 12% increase in proton conductivity over the previous nanosphere PCM-Nafion membrane. Methanol permeability was significantly decreased from 10 × 10^−7^ cm^2^/s to 4.75 × 10^−7^ cm^2^/s, symbolizing a strong electrostatic interaction between PCM and Nafion as well as a compact assembly that impedes methanol from moving across PEM. Cai et al. developed a Nafion membrane doped with sulfonic polyamide with end-capped -COOH as a bifunctional polymeric nano-sieve (BFPS) [67]. With 5% BFPS in Nafion, the proton conductivity of the composite membrane (0.31 S/cm) was about 50% higher, and the methanol permeability was reduced by 45% when compared to pure Nafion. As a result, the power output was increased by almost 30%, showing that BFPS-Nafion could be used as the PEM in DMFC.

To demonstrate the use of polyacrylonitrile in Nafion, Sigwadi et al. described a Nafion membrane reinforced with a blend of polyacrylonitrile (PAN) and zirconia oxide (ZrO_2_) [68]. Due to the enhanced water uptake but the decreased swelling ratio in the presence of PAN, a higher proton conductivity was achieved in parallel with the lower methanol permeation, which contributes to the increase in selectivity ratio. As for the polypyrrole polymer, Ben Jadi et al. reported on a study involving the coating of polypyrrole (PPy) polymer onto a Nafion membrane [69]. PPy is a conducting polymer that is applied as a barrier to methanol. According to their findings, the membrane exhibited a substantial decrease of 94% in methanol permeability after 2 h of polymerization. Proton conductivity, on the other hand, was reduced by 53% as a result of the reduced number of free sulfonate groups and the dense structure formed after modification that isolates the mobility of sulfonic acid groups. Cellulose nanocrystal (CNC) was used in a study by Hosseinpour et al. to make a modified multilayer Nafion membrane. CNC was spray-coated on one of the Nafion membrane surfaces within the stacked membranes [70]. With increasing CNC loadings up to 1.5%, the methanol permeability gradually decreased with only a slight reduction in proton conductivity. This is because the crystalline structure of CNC induces tortuosity, which interferes with the methanol diffusion pathway. However, the presence of air in the gap between the stacked membranes blocks proton migration and increases interfacial membrane resistance, lowering proton conductivity due to insufficient contact of proton pathways between the layers. Table 1 summarises the performance of polymer modified Nafion membranes in DMFCs in terms of proton conductivity and methanol permeability.

With these improved performances and features, polymer blend Nafion membranes are believed to have a high potential for use in real applications. However, before they can be used commercially, more research on optimizing the balance of proton conductivity and methanol permeability in the membrane is required, which should be considered a significant research focus for DMFC membrane in the future.

### 5.2. Adding Inorganic Filler

Adding inorganic material during the membrane preparation process is another effective and commonly used approach for improving the performance of Nafion membranes. Numerous strategies for reinforcing Nafion with different inorganic materials such as silica, metal oxides, nanoclays, montmorillonite, and zeolite have been proposed. In general, these functional inorganic fillers fall into three categories: inert hygroscopic (e.g., titania (TiO_2_) and silica (SiO_2_)), proton-conducting (e.g., ZrP), and hydrophilic with proton conductivity (e.g., functionalized zeolite) [71]. Considering that proton transfer in Nafion is still a challenge during high-temperature operation, it has been reported that the addition of hygroscopic inorganic oxides to Nafion can help maintain the relative humidity of the membrane, which can facilitate proton transport during fuel cell operation at high temperature [72]. Due to their high porosity, they are able to increase water retention in the membrane while also acting as Lewis acid sites to absorb more water [68]. Inorganic oxides also offer convincing mechanical properties to the membrane structure through the interaction between organic-inorganic components. Additionally, the incorporation of inorganic oxides results in the formation of a new membrane structure with partially filled voids, which favors methanol blocking [73]. However, the main problem with this method of inorganic filler addition is obtaining homogeneous filler dispersion throughout the polymer matrix. To tackle this issue, nanosized and high surface area fillers have been reported to be incorporated into the polymer matrix, resulting in a rigid and compact membrane [6]. The nanosized hydrophilic filler within the Nafion matrix creates an obstruction in the hydrophilic region, blocking the methanol; at the same time, the filler offers hydrogen bonding sites, enabling water retention and maintaining the mechanical strength of the membrane. However, as the filler content is increased, the hygroscopic effect gradually diminishes as the filler occupies a part of the free volume of Nafion and covers the sulfonic acid group, eventually limiting the space for proton transport. Furthermore, it is worth noting that an excess of hydrophilic fillers would result in high water uptake, serious dimensional swelling, and a decline in mechanical properties. Hence, the filler content must be optimized to acquire high performance of PEM [32].

In the work of Mazinani et al., three different hybrid membranes, which are Nafion-calcium oxide (CaO), Nafion-zirconium phosphate (ZrOH), and Nafion-CaO-ZrOH, were synthesized via the ion-exchange method for DMFC application [74]. The group found that methanol crossover was reduced dramatically for all the membranes due to the doping of inorganic particles, which hinders the transport of methanol. Proton conductivity was improved ten-fold and six-fold in Nafion-CaO and Nafion-CaO-ZrOH, respectively, when compared to Nafion. Improvement in proton conduction was attributed to the synergetic effects of water uptake and new proton hopping sites created by the ZrOH. Another approach using zirconium material to modify the Nafion membrane was to incorporate nanometer-sized hygroscopic inorganic acid, zirconium phosphate (ZrP), into Nafion via recasting or impregnation [75,76]. It was reported that there was no agglomeration observed with up to 2.5 wt% ZrP in the membrane resin. The increased proton conductivity of Nafion/ZrP was seen even at high temperatures (80 °C), owing to the hydrophilicity of ZrP and the presence of proton conductors, OH-PO_3_ in the zirconium [75]. ZrP also improved the methanol barrier properties of the Nafion membrane, where the methanol permeability of Nafion and Nafion/ZrP composite membranes is 8.84 × 10^−7^ cm^2^/s and 0 cm^2^/s, respectively, at 5 M methanol concentration and 60 °C [76]. The effect of zirconia oxide (ZrO_2_) on Nafion performance had also been investigated by the same group of researchers [77,78]. The results showed an even distribution of ZrO_2_ within the membrane, contributing to the improvement in water uptake and mechanical and thermal stability. The same group later tuned the proton conductivity of Nafion by manipulating the ionic sites by integrating sulfated zirconia oxide (S-ZrO_2_) and sulfated zirconia oxide modified with ammonia sulfate (S-ZrO_2_(NH_3_SO_4_)) [79]. The proton conductivity of the membranes increased in the following trend: Nafion/S-ZrO_2_(NH_3_SO_4_) > Nafion/S-ZrO_2_ > Nafion 117, suggesting that an increase in water uptake, the acid sites created by the sulfation, and the presence of (NH_4_)_2_SO_4_ acid in the Nafion/S-ZrO_2_(NH_3_SO_4_) membrane, all promote the formation of the ionic cluster, hence facilitating proton transport. Recently, Sigwadi et al. also published a study comparing the applicability of sulfated and phosphated zirconia nanoparticles (S-ZrO_2_ and ZrP) incorporated Nafion membrane as DMFC membranes. The results demonstrated that Nafion/S-ZrO_2_ is a more promising membrane than Nafion/ZrP in terms of proton conductivity, methanol-blocking capability, and life performance [80].

**Figure 4:**
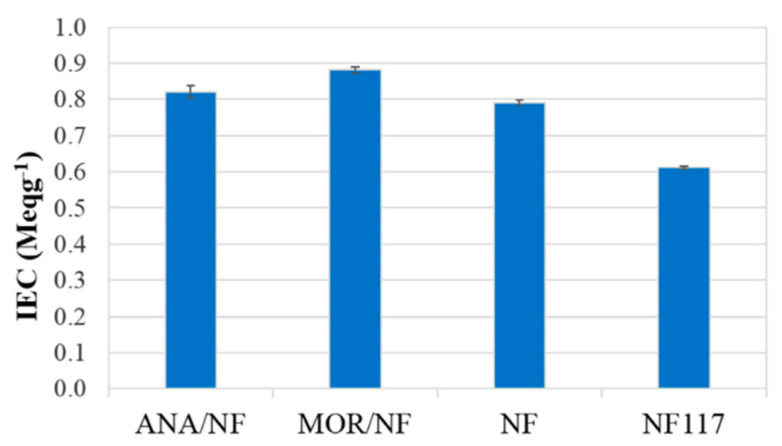
IEC of zeolite/Nafion membranes compared with recast Nafion and Nafion 117 membrane. Reprinted with permission from Ref. [83]. Copyright 2017 Elsevier.

Another inorganic component with a microporous structure, zeolite, has been extensively investigated for DMFC applications. This inorganic component is hydrophilic, ion conductive, has a high crystalline structure, and can withstand acidic or high temperatures of up to 800 °C [81,82]. Additionally, due to its higher selectivity for water over alcohol, zeolite establishes preferable sieving properties. Prapainainar et al. modified the Nafion membrane using two types of silane-modified and functionalized zeolites, modernite (MOR) and analcime (ANA) [83]. MOR/Nafion has a slightly higher proton conductivity than ANA/Nafion, which was consistent with the IEC value (as shown in Figure 4), where the IEC value indicates the concentration of accessible conductive sites, an indirect measure of proton conductivity. In view of its more impermeable characteristics, MOR/Nafion exhibited a lower methanol permeability than ANA/Nafion. This could be because MOR has a smaller pore size than ANA, allowing it to more effectively block the methanol. Taken together, these results show that MOR/Nafion has a power density 1.5 times that of ANA/Nafion and two times that of Nafion 117 in DMFC. To solve the issue of inorganic material having poor interfacial interaction with Nafion by improving their compatibility, Prapainainar et al. modified the surface of mordenite with GO and subsequently treated it with silane (S-GO-MOR) and then added it into Nafion (NF/S-GO-MOR) [84]. Due to the increased surface hydrophilic functional groups, NF/S-GO-MOR exhibited proton conductivity 1.5 times higher than those for NF/S-MOR, recast Nafion, and Nafion 117. NF/S-GO-MOR also established the lowest methanol permeability among the membranes tested, leading to a power density four-fold that of Nafion 117. In another study, Prapainainar et al. designed a Nafion/silane-modified MOR composite membrane through a solution-casting method with the use of methanol and ethanol to increase the compatibility between the filler and Nafion, improve filler dispersion and prevent void formation within the polymer matrix [81]. The ordered structure of the resulting membrane resulted in a 47% higher power density than the pristine Nafion membrane when fueled with 4 M methanol. This finding corroborates the previous study conducted by the same group of researchers [83].

**Figure 5:**
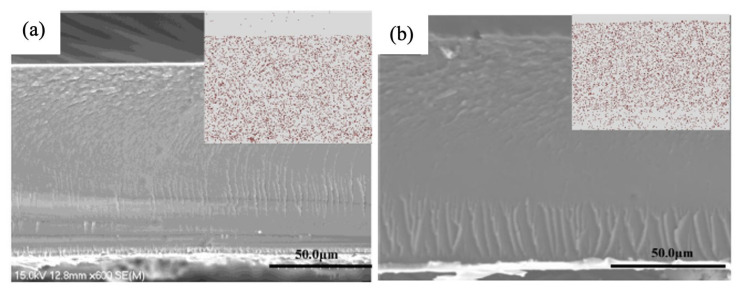
SEM images for NH_4_−X zeolite/Nafion composite membrane with 5 wt% of: (**a**) nano and; (**b**) submicron NH_4_−X zeolite. Reprinted with permission from Ref. [85]. Copyright 2015 Elsevier.

In order to selectively sieve protons and block methanol, Sun et al. used a different type of zeolite, UZM-9 zeolite, with an intermediate window size between hydrated protons and methanol to construct a Nafion composite membrane [82]. The modified Nafion membrane showed a drastic decline in methanol permeation rate, 6.845 mol/m^2^h, which was four times lower than the original Nafion membrane, resulting in a peak power density nearly 2.5-fold higher than the original Nafion. Another type of zeolite, NH_4_-X zeolite, in submicron and nanosized, was synthesized by Cui et al. and then incorporated into a Nafion membrane [85]. As shown in the SEM images in Figure 5, the 5 wt% nanosized (Figure 5a) and submicron (Figure 5b) filler dispersed uniformly within the polymer matrix, forming a dense structure. Both composite membranes exhibited a slightly higher proton conductivity than pristine Nafion, with submicron NH_4_-X zeolites composite membranes having a higher value than nano NH_4_-X zeolites composite membranes. The combination of increased proton conductivity and decreased methanol permeability in submicron NH_4_-X zeolites composite membranes results in the highest selectivity, leading to three times higher power density than the recast pristine Nafion in DMFC.

On the question of DMFC performance at relatively high temperatures (95 °C), Ercelik et al. took the first step to compare the performance of Nafion/SiO_2_ and Nafion/TiO_2_ composite membrane to that of Nafion 115 under different operating temperatures [86]. Given the hydrophilic nature of inorganic SiO_2_ and TiO_2_, both composite membranes exhibited increased water uptake and thus higher proton conductivity at high temperatures. Despite their improved performance at high temperatures, their proton conductivities were found to be less than satisfactory at low temperature. The results coincide with a study conducted by Wang et al., in which the self-crosslinked SiO_2_ with Nafion nanocomposite showed a lower proton conductivity (1 to 1.5 × 10^−2^ s/cm) than Nafion (5.02 × 10^−2^ s/cm) at 25 °C [87]. However, the maximum decrement of the methanol permeability was two orders of magnitude lower than Nafion; this resulted in a higher selectivity that suggested the membrane could be an alternative DMFC membrane. On the other hand, Yang et al. applied mesoporous silica, SBA-15, as an inorganic filler in the Nafion matrix [88]. SBA-15 has been shown to enhance water uptake and resist methanol permeation due to the filler blocking the water channel. Unlike the other silica materials discussed previously, SBA-15 was found to provide additional ionic pathways as the outer surface of SBA-15 was covered by negative charge group Si-OH, which is the active site to uptake protons, allowing all composite membranes to establish a higher proton conductivity than Nafion [18]. The SBA-15 modified Nafion membrane achieved a maximum of 80% higher power density (117 mW/cm^2^) than recast Nafion membrane (65 mW/cm^2^) and commercial Nafion 117 (96 mW/cm^2^) in DMFC.

Thiam et al. examined silica in nanofiber form doped with palladium (Pd-SiO_2_) and then coordinated with Nafion [89]. They postulated that a 3 wt% Pd-SiO_2_ nanofibers loading resulted in the highest proton conductivity of 0.1292 S/cm and the lowest methanol permeability of 8.36 × 10^−7^ cm^2^/s, giving a power density of 10.4 mW/cm^2^, which is higher than that of Nafion at 7.95 mW/cm^2^ when tested in passive DMFC. In another study, Thiam et al. incorporated a silica/silicotungstic acid (SiO_2_/SiWA) inorganic composite into Nafion [90]. According to their findings, methanol permeability was reduced by 58% compared to recast Nafion when SiO_2_/SiWA increased the tortuosity of the methanol migration channel. Unfortunately, despite the increased water uptake, proton conductivity was compromised because the ionic cluster was covered, and chain mobility was restricted as the filler content increased.

**Figure 6:**
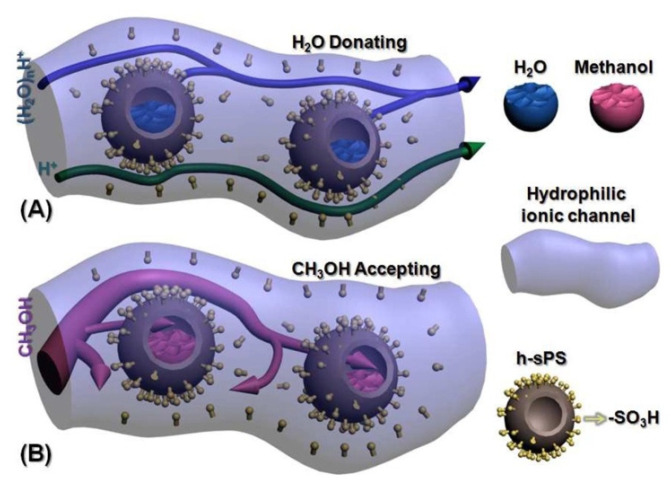
Schematic illustrations of “H_2_O donating/methanol accepting” mechanism: (**a**) releasing of free water and; (**b**) blocking of methanol. Reprinted with permission from Ref. [91]. Copyright 2015 Royal Society of Chemistry.

Functionalized silica has been widely employed to achieve a balance in the permeability of proton and methanol. Feng, Tang, and Wu prepared a Nafion nanocomposite membrane based on sulfonated SiO_2_@polystyrene (SiO_2_@sPS) through a blending-casting method [91]. When the SiO_2_@sPS content is below 2 wt%, the proton conductivity is significantly enhanced due to the interaction of additional sulfonic acid groups with the water molecules. The SiO_2_ core was etched to form a well-dispersed hollow sPS (h-sPS) sphere inside the membrane matrix, which gradually releases the free water reserved in the large interior space of the sphere (Figure 6a), further promoting proton conductivity. The fact that the methanol molecules were captured inside the sphere, together with the more twisted pathway created by the presence of SiO_2_@sPS, blocking the methanol from passing through the membrane (as illustrated in Figure 6b), results in a lower methanol permeation. On the grounds of these, the model was represented as “H_2_O donating/methanol accepting”, as provided in Figure 6.

In the light of the beneficial effects of functionalized silica on the performance of Nafion, Wang et al. impregnated Nafion with biofunctional silica (Bio-SiO_2_) to increase proton conductivity [92]. Four different amino acids (cysteine (Cys), serine (Ser), lysine (Lys), and glycine (Gly)) were grafted onto Bio-SiO_2_ to provide proton exchange sites and proton-conducting pathways. Amino acids act as both proton donors and acceptors, offering higher proton conductivity. Among the four samples, Nafion-Cys stood up in terms of proton conductivity (0.2424 s/cm); however, it also displayed the highest methanol permeation, which was reasonable given its higher water uptake. Despite this, Nafion-Cys demonstrated a higher methanol resistivity when compared to Nafion, which is attributed to the formation of an anfractuous barrier network and tortuous diffusion channel by SiO_2_ in the membrane matrix coupled with the hydrogen bonding formed between the filler and Nafion. Additionally, the Bio-SiO_2_ filler balanced the relationship between swelling and water uptake, contributing to a high degree of dimensional stability. A further two forms of functionalized silica were investigated by Wang et al., who successfully built proton-conductive membranes by immersing a PVdF porous membrane in the mixed suspension containing Nafion and SiO_2_ nanospheres functionalized with –NH_2_ and –COOH, respectively, to form PVdF/Nafion/SiO_2_–NH_2_ and PVdF/Nafion/SiO_2_–COOH [93]. PVdF helped to reduce swelling ratios at high water uptake by providing excellent mechanical stability. SiO_2_ grafted with –NH_2_ and –COOH provided pathways for proton hopping and formed a continuous transportation channel, which aided in efficient proton migration. Additionally, SiO_2_ increases the tortuosity of the composite membranes, resulting in lower methanol permeability. These improvements increased selectivity and confirmed the feasibility of using functionalized silica in DMFC membranes.

Titania (TiO_2_), inorganic particles, can also be functionalized prior to being introduced into Nafion, as described by Cozzi et al. [94]. The sulfonic acid group in the functionalized filler, TiO_2_-RSO_3_H, provided new acidic sites for proton transfer over a large range of temperatures. However, the methanol permeability of the composite membrane (0.75 × 10^−7^ cm^2^/s) was slightly lower than the unfilled Nafion membrane (1.05 × 10^−7^ cm^2^/s). Despite the unfavorable methanol-blocking properties, the feasibility of functionalized TiO_2_ in DMFC was proven by a 40% improvement in power density. Taking virtues of TiO_2_, Allodi et al. sought to demonstrate the homogeneity of nanosized sulfated TiO_2_ (S-TiO_2_) used as a filler in Nafion-based PEM [95]. The Raman spectra revealed that at filler concentrations of 5% and 7%, S-TiO_2_ was present throughout the membrane surface but was not homogenously distributed. Despite its uneven distribution, S-TiO_2_ contributes to the formation of a continuous proton percolation path, which is predominant for proton transportation.

Inorganic additives such as tin oxide (SnO_2_), cerium oxide (CeO_2_), and aluminum oxide (Al_2_O_3_) have also been added into Nafion to prepare a variety of composite membranes with enhanced performance. For instance, Scipioni et al. delved into the tailoring of the Nafion membrane using sulfated nanosized tin oxide (SSnO_2_) prepared via sol-gel synthesis from two different precursors: Sn (II)-2-ethyl-hexanoate and aqueous hydrolysis of tin (IV) chloride (SnCl_4_) [96]. The smaller crystallite size of SSnO_2_ synthesized from SnCl_4_ resulted in a larger surface area, which led to higher water uptake, a more uniform organization, and increased mechanical strength. A bifunctional hygroscopic metal oxide, cerium oxide (CeO_2_), was studied by Velayutham, Sahu, and Parthasarathy on its effect on the Nafion membrane for DMFC [6]. The authors claimed that adding CeO_2_ covered the hydrophilic channels and increased the surface roughness, which in turn improved contact with the electrodes. Incorporating 1 wt% of CeO_2_ in Nafion results in a 30% increase in proton transport compared to pristine Nafion. This is because the inclusion of this hygroscopic filler increases water uptake. Additionally, it was found that CeO_2_ reduced the size of the channel in the composite membrane, hence reducing methanol crossover. Cerium oxide is also used as a radical scavenger; however, this is a lesser-known application. Cerium is an effective free radical scavenger due to its oxidation states (+3 and +4), which ease the reversible redox reaction between Ce^3+^ and Ce^4+^ [97,98]. Furthermore, free radicals react faster with cerium than with ionomer membranes, which reduces the degradation rate of the membrane [29]. This was established by Weissbach, Peckham, and Holdcroft in their study [99]. They examined composite Nafion with 10 wt% of CeO_2_ and found that Nafion/CeO_2_ reduced mass loss, fluoride release, and loss of sulfonic acid groups, which indicates fewer signs of degradation.

**Figure 7:**
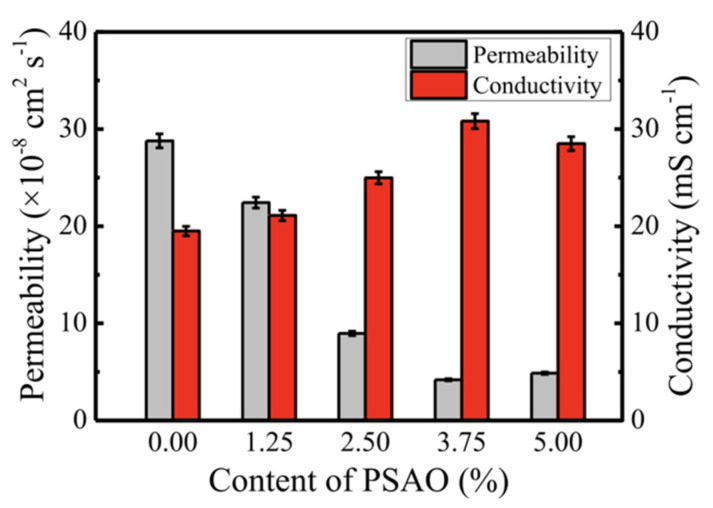
Methanol permeability and conductivity of pristine Nafion and PSAO−based Nafion composite membranes. Reprinted with permission from Ref. [101]. Copyright 2018 Elsevier.

In one of these studies, aluminum oxide (Al_2_O_3_) was grown using trimethylaluminum (TMA) as a precursor and coated on Nafion, which provides increased mechanical strength [100]. Al_2_O_3_ was found to fill the polymer structure, forming a more rigid membrane that is more resistant to water absorption and swelling. However, this resulted in a drop in proton conductivity, despite the coated membrane impeding methanol permeability by about 30–50% lower than Nafion 115, depending on the operating conditions. In order to eliminate the trade-off between proton and methanol transport, Cui et al. prepared an acid-functionalized porous silicon aluminum oxide (PSAO) Nafion-based composite membrane through the solvent recasting method [101]. The PSAO successfully improved the proton conductivity of the membrane compared to the pristine Nafion (as presented in Figure 7), owing to an increase in water uptake and IEC. The PSAO, accompanied by the existing Bronsted acid sites −Si-OH and −Si-O-SO_3_H, aided in coordinating proton transport. Moreover, the methanol permeability of the composite membrane was reduced due to the crosslinking between sulfonic groups and PSAO, forming a compact membrane and thus narrowing the transport pathway. PSAO, by forming hydrogen bonds with methanol, created a tortuous channel and delayed the movement of methanol. As a result, DMFC using a Nafion-PSAO membrane attained a peak power density four times that of pristine Nafion.

**Figure 8:**
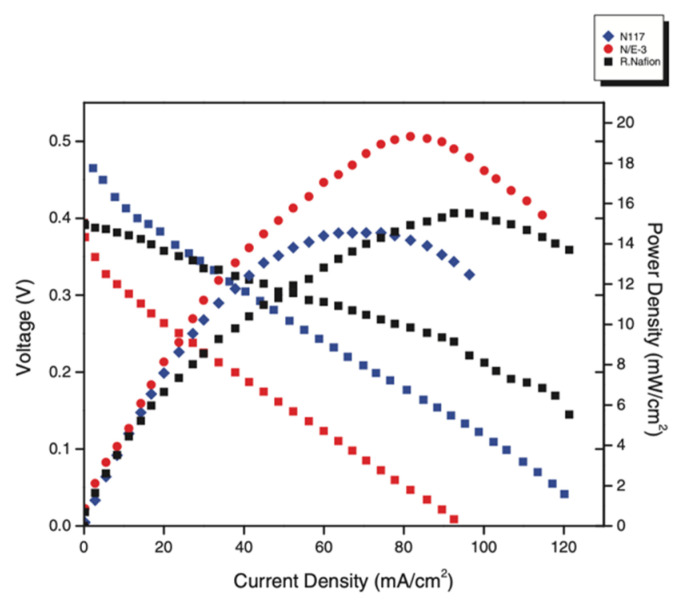
Polarization curve of Nafion 117, Nafion/eggshell composite membrane, and recast Nafion membrane at room temperature. Reprinted with permission from Ref. [102]. Copyright 2020 Wiley.

The trending concept of using waste material as a filler has also been introduced into the modification of Nafion membranes. Hamid, Kamarudin, and Karim modified Nafion with eggshell powder, which is known to have high content (about 93%) of calcium carbonate (CaCO_3_) and to be hydrophilic [102]. The casted Nafion/eggshell composite (N/E-3) membrane with a filler content of 3 wt% exhibited a higher proton conductivity of 0.2414 S/cm and a lower methanol permeability of 8.40 × 10^−7^ cm^2^/s, resulting in a higher power density of 19.34 mW/cm^2^ in DMFC (as shown in Figure 8). The composite membrane also showed improved thermal stability and tensile strength. Montmorillonite (MMT), also known as clay, is another effective inorganic filler for Nafion. It is a protonic conductor with ionic conductivities of 1 × 10^−4^ S/cm and is composed of repeating triple layers of alumina sandwiched between two layers of silica. According to Felice, Ye, and Qu, the Nafion-MMT nanocomposite membrane provides a longer tortuous path for methanol penetration, effectively retarding the methanol crossover [103]. However, Nafion-MMT has a significantly lower proton conductivity than Nafion. A step was taken by Azimi and Peighambardoust to boost the proton conductivity of Nafion-MMT by mixing cesium salt of heteropolyacids (CsPW) with MMT [104]. Hydrophilic CsPW enhances water uptake, while more acid groups in CsPW provide additional IEC for proton migration. By creating a tortuous microstructure, MMT improved the methanol barrier performance to 1.651 × 10^−6^ cm^2^/s, compared to 2.078 × 10^−6^ cm^2^/s for plain Nafion. Table 2 summarizes performance in DMFC based on the proton conductivity and methanol permeability from inorganic material modified Nafion membranes.

In summary, the addition of inorganic particles to Nafion for membrane preparation can be considered an effective strategy for improving membrane performance. However, it is vital to keep an eye out for two factors that could affect the membrane performance: inorganic fillers concentration and the functionalization of additives. Functionalization, or modification with proton exchange groups, is believed to be an effective approach for overcoming the trade-off between the benefits and drawbacks of inorganic filler in Nafion.

### 5.3. Adding Ionic Liquid

**Figure 9:**
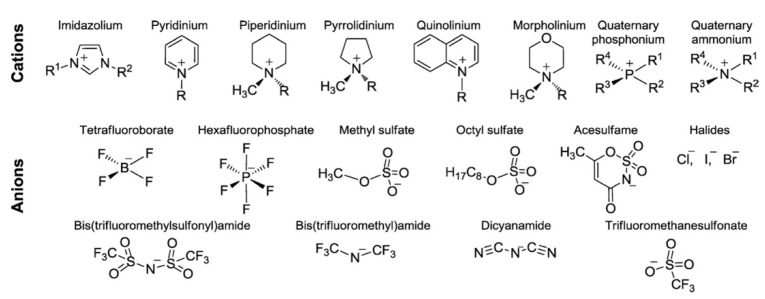
Structure of commonly used cations and anions in ionic liquid. Reprinted with permission from Ref. [111]. Copyright 2017 American Chemical Society (United States).

Ionic liquid incorporation is a successful way of maintaining the inherent proton conductivity of Nafion at high temperatures, which has attracted a great deal of attention recently. IL is a molten organic salt at room temperature with a melting temperature of less than 100 °C [105]. IL is made up entirely of ions and is composed of a combination of an organic cation and either an organic or inorganic anion. Figure 9 illustrates the commonly used cations and anions. The tunability of IL through the use of different cation or anion pairs contributes to its flexible solvation features [106]. In addition, the high ion mobility of IL favors its usage as an electrolyte [107]. It was reported that Nafion incorporated with IL has a high proton conductivity, which is attributed to the protic elements (active protons available at the cation) in IL. Particularly, the nitrogen sites in imidazole-based IL form hydrogen bonds with the sulfonic group of Nafion, allowing for facile proton hopping in systems with low water content [108,109]. Additionally, because of its negligible low vapor pressure, IL does not readily evaporate and thus has shown promise as an alternative strategy for the creation of low humidity and high-temperature PEM materials [110].

Despite the advantages feature of IL, research on the IL-modified Nafion membrane is limited, with the majority of related work focusing on hydrogen fuel cells and less on DMFC. One of the related studies was conducted by Neves, Coelhoso, and Crespo [112]. They investigated the effect of different types of IL cations on methanol crossover by incorporating them in Nafion 112 at varying degrees of incorporation. As shown in Table 3, methanol crossover was reduced by 60 to 600 times for various IL cations incorporated in Nafion 112. The electrostatic interaction between the cation in IL and the sulfonic acid group in Nafion resulted in a more structured organization, which could account for the significant decrease in methanol crossover. Additionally, increased water uptake was noticed at a higher concentration of the IL-modified Nafion membrane as the cation from IL replaced H^+^ in protonated form of Nafion, which contributed to an improvement in the solvation of water molecules. Thus, water became more contained and structured within the composite membrane, which brought larger methanol diffusion resistance. However, the proton conductivity of the composite membrane was not investigated in the study to confirm its applicability. Later, Yang et al. used a self-assembly technique to incorporate IL cation 1-butyl-3-methylimidazolium (BMIm) into Nafion, which was then doped with phosphoric acid (PA) [113]. The IL-modified Nafion membrane reduced methanol crossover by two orders of magnitude compared to Nafion 115. Additionally, the composite membrane demonstrated suitable conductivity under conditions of no humidity and high temperature as PA supplied hydrogen bonding for proton transport through the hopping mechanism. It was also found that PA helps to improve thermal stability and maintain mechanical strength.

In short, ionic liquid has been proven to be a suitable proton conductor. It is important to choose a suitable ionic liquid that is compatible with Nafion to reduce the trade-off between proton conductivity and mechanical stability. In addition, an emphasis should be placed on minimizing long-term stability issues with ionic liquid in Nafion, such as PA leakage, toward achieving desirable membranes for DMFCs.

### 5.4. Incorporating Carbon Nanomaterials

**Figure 10:**
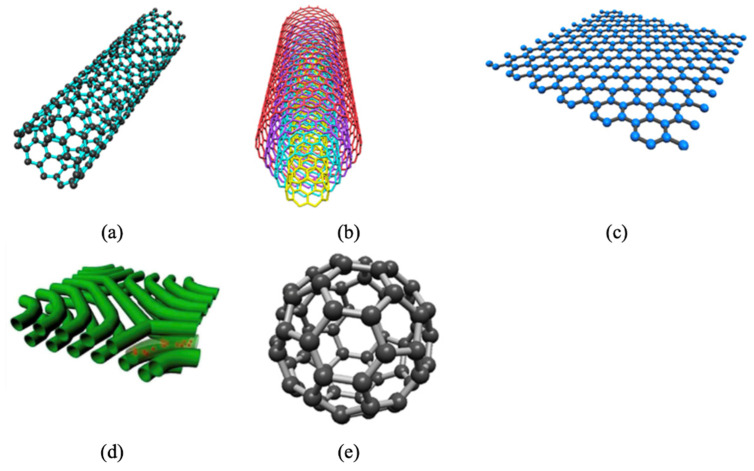
Different types of carbon nanomaterial such as (**a**) SWCNT; (**b**) MWCNT; (**c**) graphene; (**d**) mesoporous carbon; (**e**) fullerene. Reprinted with permission from Ref. [114]. 2013, Elsevier; Reprinted with permission from Ref. [115]. Copyright 2013 American Chemical Society (United States).

Although carbon nanomaterials are classified as inorganic materials, due to their widespread use in DMFC membranes, this section explicitly highlights the properties and performance of composite membranes incorporated with carbon-based nanomaterials. Carbon nanomaterials, which include carbon nanotube (CNT) (Figure 10a,b), graphene (Figure 10c), mesoporous carbon (Figure 10d), and fullerene (Figure 10e), have been considered promising fillers and reinforcing additives for Nafion owing to their distinctive structure and physical properties that contribute to methanol-blocking characteristic and mechanical strength [33]. 

However, these heterogeneous fillers are difficult to disperse uniformly within the polymer matrix due to Van der Waals forces that limit the interfacial interactions, resulting in aggregation, poor mechanical stability, and altered transport efficiency. Additionally, the filler reduces the density of the sulfonic acid groups [116]. As a result, if excess filler is inserted, the proton conductivity of the membrane is frequently decreased. Thus, to improve their dispensability, carbon nanomaterials can be grafted with functional groups, including chitosan or silica, but mostly acids, on their surface to improve the interfacial interaction between functionalities and polymer and thus achieve homogeneous distribution [33].

Carbon nanotubes (CNTs), a well-known material with a unique tubular structure of nanoscale diameter and micrometer length, have been extensively explored as an additive in the fabrication of Nafion composite membrane. CNTs can appear multi-walled or single-walled. The single-walled carbon nanotube (SWCNT) (Figure 10a) structure is more ordered and exhibits excellent flexibility. However, a multi-walled carbon nanotube (MWCNT) (Figure 10b) is more suitable for incorporation into PEM since it has lower electronic conductivity and higher surface defects than SWCNT. Thus, functionalities can be added to the surface of MWCNT more easily. It has been demonstrated that the inclusion of CNT into the Nafion matrix reduced the methanol permeability, but the effect of CNT filler separated the Nafion chain and impeded the proton conductivity [117]. In this respect, CNT was decorated with 0.5 wt% of hydrophilic chitosan (CS), forming hydrogen bonds between CS and Nafion as well as non-covalent interaction between CS and CNT [117]. By improving the interfacial interactions and solubilization of CNT in Nafion, it provided a more pronounced reduction in methanol permeability to 2.03 × 10^−7^ cm^2^/s, which is one order of magnitude decrease compared to Nafion 117. CS in the hybrid membrane, at the same time, contributes to the Grotthuss proton transport mechanism by retaining water molecules even at high temperatures. Moreover, the homogeneous dispersion of CS-CNT in Nafion prevented CNT from re-bundling and agglomeration. The authors also noted that keeping the CNT content below the percolation threshold of 2 wt% may prevent charge transfer across the membranes and minimize the risk of forming a short circuit in the membranes. On the other hand, Molla-Abbasi, Janghorban, and Asgari synthesized CNT/Nafion and coated it with 1 wt% of silica as well as phosphotungstic acid (PWA) to produce a Nafion-inorganic-inorganic compound [118]. A higher ion exchange capacity and water uptake were shown in the CNT/SiO_2_/PWA composite Nafion membrane. This is because PWA acts as an immobilized acid proton conductor via electrostatic interaction with SiO_2_, while the hydroxyl group of PWA improves the water absorption rate, which has been proven to improve proton conductivity at temperatures over 80 °C.

**Figure 11:**
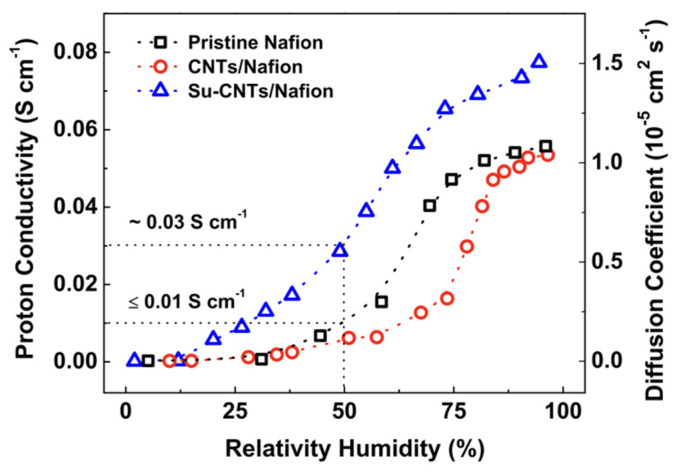
Proton conductivity and proton diffusion coefficient of membranes as a function of relative humidity. Reprinted with permission from Ref. [119]. Copyright 2018 American Chemical Society.

**Figure 12:**
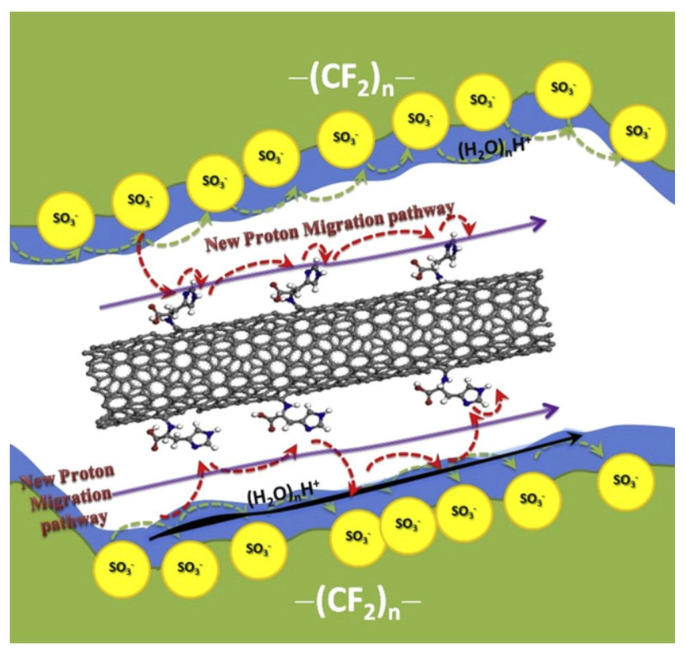
Interaction between nitrogen groups of imidazole−functionalized CNT and H^+^. Reprinted with permission from Ref. [108]. Copyright 2013 Elsevier.

Functionalization is one of the strategies proposed for improving the dispersion of carbon nanomaterials in the polymer matrix, hence enhancing membrane performance. Sulfonated CNT with sulfonic acid functionalities was a successful attempt [119]. The sulfonated CNT/Nafion (Su-CNTs/Nafion) presented superior proton conductivity to pristine Nafion and CNT/Nafion even at low humidity, as shown in Figure 11. This is because sulfonated CNT boosts the growth of interconnected ionic-water clusters and aids in binding water molecules. Furthermore, Su-CNTs prevented the thermal distortion of the proton-conducting network and improved the mechanical properties of the membrane. Considering the electrical conductive characteristics of CNT, the electronic conductivities of Su-CNTs/Nafion were measured and found to be nearly identical (1.1 × 10^−6^ s/cm) to those of the pristine Nafion membranes. This could be because the surface functionalization of CNT was carried out under highly oxidizing conditions, and the associated oxidative defects result in electrical resistance. CNT can also be functionalized with imidazole-containing materials, such as ionic liquid, to localize proton carriers within the membrane matrix and overcome the retardation of proton conductivity induced by CNT [108]. In light of this, histidine was used as an imidazole-based amino acid to modify CNTs and disperse them in Nafion through solution casting. The methanol crossover of the fabricated nanocomposite membrane was found to be lower than that of Nafion, which was attributed to the lower water uptake. The strong acid-base interaction between the sulfonate groups in Nafion and the imidazole group on CNT impacted the water absorption by altering the size of the nanochannel. Reduced water uptake prevents PEM from excessive swelling and depressing the mechanical strength. On the other hand, the proton conductivity was improved since the lone free electrons of the nitrogen groups in the imidazole material formed hydrogen bonds with protons (H^+^), thereby enhancing Grotthuss-type proton transport, as shown in Figure 12. These findings resulted in a higher power density produced by DMFC.

Graphene is another class of carbon nanofillers with a two-dimensional structure and a single atomic layer thickness [120]. Graphene has been widely studied in various fields owing to its intriguing thermomechanical stability and large number of exposed active sites. A perfect graphene monolayer is impermeable to all atoms and molecules but highly permeable to protons [121], making it an excellent material for fuel cell membranes. Graphene can be prepared through a promising and economic route by oxidizing graphite and then exfoliating it. The resulting graphene oxide (GO) can be further reduced to eliminate most of the oxygen-containing moieties on its surface to obtain reduced graphene oxide (rGO). Thanks to the oxygen-containing groups (carboxyl, hydroxyl, and epoxy groups) and the large surface area of GO, its hydrophilic properties are enhanced, and more reaction sites are available for further functionalization to improve proton conductivity [33]. In addition, GO was among the materials studied for its ability to suppress methanol crossover.

**Figure 13:**
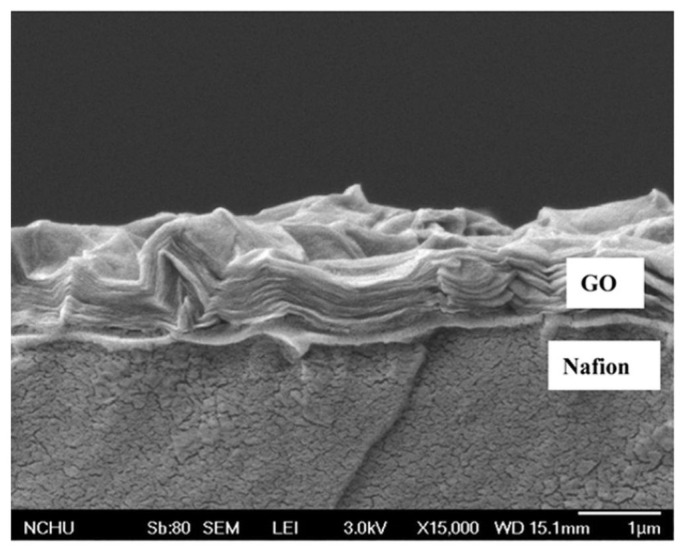
SEM image of the GO−laminated Nafion membrane. Reprinted with permission from Ref. [122]. Copyright 2013 Elsevier.

**Figure 14:**
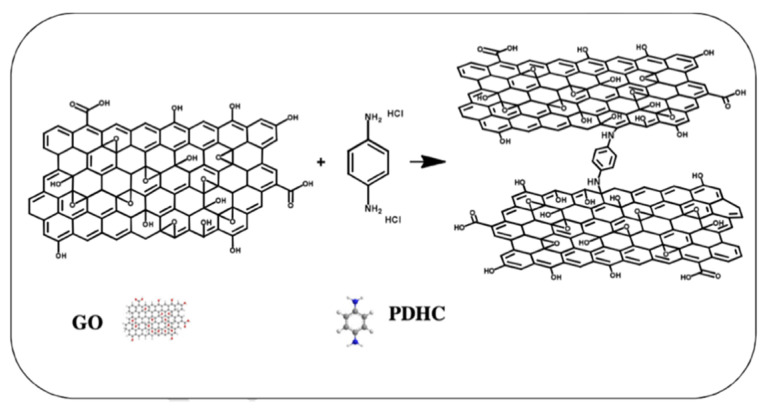
Schematic illustration of reactions between GO and PDHC. Reprinted with permission from Ref. [123]. Copyright 2015 Elsevier.

Graphene may also be found in a multilayer form, and a few research papers have shown that these multilayer and highly ordered nanostructures can withstand the negative effect of larger-sized filler particles [1]. Based on this concept, Lin and Lu explored a dual-layer membrane by laminating GO paper onto a Nafion membrane for DMFC at high methanol concentration [122]. As shown in Figure 13, the parallel alignment of GO remains intact within the laminate membrane and adheres well to Nafion. Methanol crossover was reduced by 70% when compared to the Nafion membrane; however, the composite membrane lost 22% of proton conductivity. Nevertheless, the adverse effect on proton transport is offset by the barrier effect of GO, which results in a selectivity increase of 38% and 100% at 6 M and 8 M methanol concentrations, respectively. Wang et al. developed a novel approach for preparing a multilayer PEM by laminating GO nanosheets on the surface of recast Nafion and crosslinking with 1, 4-phenyldiamine hydrochloride (PDHC) [123]. When compared to recast Nafion, the composite membrane significantly reduced methanol crossover by two orders of magnitude. This is explained by the formation of chemical bonds between PDHC and GO (Figure 14), which tightly hold the structure and prevent methanol from passing through the water-filled channels. As with the previously described study, the decrement in methanol permeability counterbalanced the decrease in proton conductivity; therefore, the composite membrane showed an increasing trend in selectivity. Yan et al. reported another example of a multilayer composite membrane with a superior methanol barrier [124]. The multi-layered membrane structure, which consisted of an inner monolayer of graphene and two thin Nafion membranes on the outside, significantly suppressed methanol permeation by 68.6% relative to the pristine Nafion membrane. The proton conductivity of the sandwiched membrane was observed to be 29% and 7% lower than that of the Nafion membrane at temperatures of 25 °C and 80 °C, respectively. Nevertheless, the intrinsic sieving effect of the graphene layer increased the selectivity of the membrane, leading to a higher performance of the DMFC with a larger power density than pristine Nafion at low or high methanol concentration.

As previously discussed, incorporating GO in Nafion membranes reduces proton conductivity due to a decrease in sulfonic acid density. To compensate for the loss of conductivity, graphene is functionalized with proton-conducting groups such as sulfonic acid, which is proton conductive. As demonstrated in a study by Feng and co-workers, a Nafion-based PEM incorporated with sulfonated graphene oxide-silica (S-GO-SiO_2_) nanohybrid particles exhibit improved proton conductivity due to the increased water uptake for solvating protons and the presence of additional sulfonic acid groups [22]. Surprisingly, there was no obvious increase in the swelling of the composite membrane after immersion in either water or methanol. Furthermore, increasing the content of S-GO-SiO_2_ nanoparticles rendered a depressing effect on methanol crossover, which implied a selectivity of two orders of magnitude higher than that of recast Nafion. Another successful effort to improve the proton conductivity of graphene incorporated membrane was performed by Parthiban et al., who introduced graphene that had been functionalized with sulfonic acid-containing aryl radicals in Nafion [125]. As expected, the additional sulfonic acid groups per unit volume serve as ionic exchange sites and significantly increase water uptake for both the hopping mechanism and solvating protons for migration. Accordingly, the proton conductivity increased from 65.3 mS/cm to 104.0 mS/cm when graphene content raised from 0 wt% to 1 wt%. Furthermore, methanol was successfully blocked by the 1.5 wt% of filler, which is a 50% reduction over pristine Nafion. The collaboration of these improvements confirmed Nafion-sulfonated graphene as a potential candidate for achieving high power density in DMFC application.

Several studies have examined a similar concept based on functionalized GO but with a different membrane fabrication method. Tsai et al. coated SGO and sulfonated-activated carbon (SAC) onto Nafion membranes repeatedly [126]. Their findings showed that the highly porous SAC aided in water retention while SGO restricted methanol permeation due to its higher selectivity toward water over methanol. Compared to bare Nafion, a 10% increase in power density could be observed by applying bilayer SGO and SAC-modified Nafion membrane in DMFC. Another study was slightly different from the previous attempt, in which GO was modified with poly (diallyldimethyl ammonium chloride) (PDDA) and grafted on the surface of a Nafion membrane through a layer-by-layer approach [127]. The positively charged PDDA successfully formed the first layer binding to the Nafion with a negative charge on the surface. PAAD-Nafion was then dipped into the negatively charged GO and resulted in a dense and uniform Nafion-PDDA-GO multilayer membrane. The resultant multilayer membrane has a 67% lower methanol permeability than pristine Nafion and a much lower cathode oxidation current density, indicating a reduction in methanol crossover. Li et al. used the swelling-filling (SF) strategy to insert bifunctional sheared GO into Nafion [128]. This approach improved the membrane, specifically by reducing methanol permeability by more than 70% while raising proton conductivity by 26%. It is worth mentioning that the SF-treated membrane does not destroy the proton-conductive channel, which might occur in a traditionally modified membrane. With these improved properties, the SF-treated SGO-Nafion membrane proffered 50% higher power output than the pristine Nafion membrane in DMFC applications.

Carbon dots (CDs) have recently appeared as carbon nanomaterials with a diameter of less than 10 nm. CDs can be synthesized via pyrolysis of citric acid, which is considered a low temperature and economic pathway. CDs can be easily functionalized by blending them with other types of fillers or polymers to alter their functions for specific applications. Jia, Tang, and Wu blended Nafion-modified CD (NCD) with Nafion to form a composite membrane with carboxyl and sulfonic acid functional groups [129]. The multifunctional groups interact with Nafion through hydrogen bonding, forming a suitable stability composite membrane that could be used at higher temperatures (80 °C and 100 °C). A significant increment in proton conductivity of five-fold was reported, and the methanol permeability was found to be 50% lower than that of recast Nafion, indicating that NCD contributes to the improvement of the membrane. Another carbon nanomaterial, fullerene (C_60_), was also explored as a potential additive in Nafion. Although the bulky size of fullerene makes it difficult to disperse homogenously in Nafion, its high surface area and suitable mechanical and chemical stability are added advantages. It was pointed out that fullerene acts as a radical sponge and retards the degradation of Nafion since it reacts more readily with low molecular weight radicals by adding the radicals to the carbon-carbon double bonds [130]. In the study of Rambabu, Nagaraju, and Bhat, in which fullerene was functionalized with a sulfonic acid group (FF) to facilitate proton conduction by creating a more connected proton transport channel [131]. The Nafion-FF composite membrane with 1 wt% of FF loading delivered better power output than recast Nafion in DMFC. This is ascribable to an improved methanol barrier effect, and the increased proton hopping site allows proton transfer to be 32% higher than recast Nafion.

In summary, functionalized CNT contributed significantly to the improvement of the mechanical strength and ionic conductivity of PEM, while sheet-like functionalized GO efficiently controlled the methanol permeability through Nafion by capturing the methanol. Fullerene has not been widely investigated as a modifier for Nafion; however, it may provide the basis for a new type of tailored ionic conductor. It is also worth noting that, as proven by many studies [61,93,94,125], the functional groups on carbon nanomaterials play a crucial impact on the performance of DMFC membranes, making this a promising avenue for future studies.

## 6. Other Structural Modifications

Ion implantation is a technique for physically modifying membrane surfaces without affecting their bulk properties. However, this approach has received little attention in the field of PEM preparation. In a study by Lee, et al., in which light ion implantation through irradiation was used to modify the surface of Nafion [132]. During irradiation, the accelerated H^+^ ions will be implanted into Nafion, and the high energy irradiation will break the chemical bonds on the Nafion surface, causing scission of the hydrophilic side chains of Nafion and production of radicals. After being oxidized by oxygen in the air, these radicals form hydrophilic functional groups such as carboxyl (−COOH), carbonyl (C=O), and hydroxyl (−OH). The generated hydrophilic groups are capable of maintaining the water retention ability of Nafion and contributing to its proton conductivity. Furthermore, it was speculated that the hydrophilic groups formed would have less affinity toward methanol. Apart from that, the ion-implanted membrane has a smaller hydrophilic channel, making it more difficult for methanol to get through, resulting in a 58% reduction in methanol permeability compared to the original Nafion.

**Figure 15:**
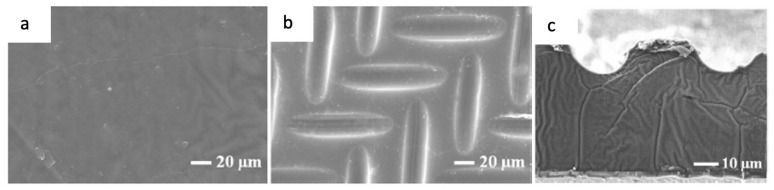
SEM image of (**a**) unmodified; (**b**) and (**c**) hot−mold−modified of Nafion membrane. Reprinted with permission from Ref. [133]. Copyright 2017 Elsevier.

Another option for altering the microstructure of the Nafion membrane is to use a simple hot-mold-modifying method (h-Nafion), in which the membrane was hot-pressed between a stainless steel sheet and a metal mesh mold at 3 MPa under 135 °C for 6 min, as proposed by [133]. From the SEM image (Figure 15b,c), the spindle-type and consistent size groove arrays are uniformly distributed on the surface of the h-Nafion membrane, in contrast to the smooth surface of the Nafion 212 membrane (Figure 15a). The resulting h-Nafion membrane has a higher compaction degree (as shown in Figure 15b,c), which is ideal for improving the contact area between the membrane and the electrode, thereby increasing charge/discharge rate and decreasing impedance while also lowering the methanol crossover. The modified membrane established a lower swelling degree than Nafion 212 membrane, resulting in 31.9% higher dimensional stability. Correspondingly, a 13.3% increase in discharge power density of DMFC was achieved by the modified Nafion membrane.

An attempt to compare two differently processed but chemically identical (same chemical composition and equivalent weight) Nafion membranes was published by Ling et al. [134]. The researchers pointed out that the membranes fabricated via two different routes, extrusion and solution casting, would exhibit significantly different physicochemical properties, such as the structure of the water channel and the chemical interaction between water and Nafion, all of which are intimately linked to the performance of DMFC. The diameter of the water channel in the extruded Nafion membrane is smaller, which favors the accommodation of non-bulk water (water molecules that are in contact with hydrophilic sulfonic groups of Nafion). Additionally, it is found that extruded Nafion membrane may transport water and protons more rapidly than solution-cast Nafion membrane. As a result, extruded membrane showed higher proton conductivity compared to the solution-cast membrane.

Furthermore, it was discovered that altering the size of the ionic cluster by exposing the Nafion membrane to ultraviolet (UV) radiation or supercritical carbon dioxide (Sc-CO_2_) effectively suppressed the methanol crossover effect. UV radiation provides crosslinking in Nafion, forming a dense network that restricts the diffusion of methanol molecules. UV radiation also enhances the ionic interaction between the sulfonic groups, which results in the formation of continuous hydrophilic channels that allows proton hopping, thus enhancing the proton conductivity. The optimally UV exposed membrane showed a significant improvement in voltage as well as power density by a factor of 1.2 to 1.5 [135]. On the other hand, the Nafion membrane pre-treated with Sc-CO_2_ exhibited a lower methanol permeability than that of the as-received Nafion 212 membranes [136]. This is because the crystallite size increases after Sc-CO_2_ treatment, which would act as stable physical crosslinks to suppress methanol transport. Additionally, the higher crystallinity improves the mechanical strength and dimensional stability of the membranes. In addition, the water is strongly bound to the ionic group in the treated membrane, thus facilitating proton transport. More interestingly, the treated Nafion 212 membranes were thinner than the original Nafion membrane, lowering the resistance to proton transport.

Table 4 summarizes some of the other modifications made to Nafion. A number of Nafion-based composite membranes have shown compelling performances, indicating that Nafion improvement may be achieved effectively. Despite the fact that some approaches can improve the methanol resistance of Nafion, they may also have an impact on other membrane parameters such as proton conductivity and mechanical strength. Hence, further research is required to find a balance between the various features of DMFC membranes in order for the modified membranes to be commercialized.

## 7. Challenges and Future Prospects

Suitable performance of polymer electrolyte membrane in DMFC must meet two important requirements: high proton conductivity and low methanol permeability. Despite commercially available Nafion membranes, the most extensively used membrane in DMFC, have many advantages, their vast applications still suffer from a high crossover rate of methanol fuel. To overcome the methanol crossover issue, many researchers have paid attention and efforts to develop composite membranes by adding inorganic fillers, carbon nanomaterials, or low-alcohol compatibility polymers into Nafion. Unfortunately, inorganic filler and carbon nanomaterial have low proton conductivity and are incompatible with Nafion, resulting in non-uniform dispersion within the Nafion polymer matrix. These considerations prompted the functionalization of the filler through sulfonation by grafting proton-conducting groups in order to achieve the desired proton conductivity and homogeneity of the composite matrix. However, functionalization needs careful inspections since the degree of sulfonation and the resulting hydrophilic functional groups affect the water content and mechanical strength of the membrane. Another approach is to incorporate the ionic liquid into Nafion, which not only makes it more tolerant toward the effects of high temperatures but also improves proton conductivity. On the contrary, ionic liquid deteriorates the mechanical strength of the membrane. Therefore, there is a need to develop alternative additives or more functionalization routes to optimize the methanol-blocking property, water uptake, mechanical strength, and proton conductivity of the modified Nafion membrane.

Another limitation of Nafion membranes is their high production cost. As a result, the amount of Nafion in the modified membrane should be kept to a minimum by incorporating modifiers or thinning the membrane to decrease the cost of DMFC. In addition to Nafion, other types of base polymer membranes (e.g., non-fluorinated hydrocarbon) with advantages such as lower methanol uptake and high proton conductivity can be considered in the bid to reduce the cost of the membrane. Long cycle life is also a challenge in practice, despite the fact that a large number of modified Nafion membranes have provided promise in reducing methanol permeability and/or improving proton conductivity. The durability of the modified membranes should thus be the subject of more research in light of their practical use.

## 8. Conclusions

In this article, some promising possibilities for modifying Nafion membranes have been discussed, including the use of inorganic materials, carbon nanomaterials, polymers, ionic liquids, and other structural developments to circumvent the shortcomings of Nafion in DMFC application. Inorganic fillers and carbon nanomaterials are often endowed with blocking effects by creating tortuous or zigzag pathways that hinder the passage of methanol from anode to cathode. Thus, a higher methanol concentration at the anode feed can be used to increase DMFC output. This approach also improves the mechanical properties and thermal stability of the membrane. However, the aggregation and incompatibility of inorganic fillers within the polymer matrix, which results in a decrease in DMFC performance, is a critical concern that must not be overlooked. Functionalization of inorganic materials with proton-conducting groups such as sulfonic acid and oxygen-containing groups has been demonstrated to be a useful way of improving the interaction between the filler and the polymer matrix. Apart from filler inclusion, Nafion and other polymers can be blended to increase the selectivity of the membrane. However, future research is needed to optimize the balance between proton conductivity and methanol permeability in the membrane. Worth noting that the incorporation of ionic liquids into Nafion may tackle the conflict between proton conductivity and methanol crossover; however, their viability requires further investigation due to limited research. The identification of the various obstacles that existing approaches face provides some insight into further research aimed at improving the performance of a PEM. Moreover, with the expansion of the range of available materials, continued advancements on PEM with desired features for DMFC such as high mechanical and chemical sustainability, water retention, proton conductivity, low methanol crossover, inexpensive as well as environmentally friendly membrane should be included in the future studies.

## Figures and Tables

**Table 1:** Summary of the performance of polymer modified Nafion membrane based on proton conductivity and methanol permeability.

Modified Nafion Membrane	Filler Content (wt%)	Test Condition Temperature (°C)	Proton Conductivity (mS/cm)	Methanol Permeability (cm^2^/s)	Reference
SDF-PAEK@Nafion	15	80	↑ (197)	↓ (2.03 × 10^−6^)	[48]
Nafion/PAni	-	90	↓ (10.66)	↓ (7.71 × 10^−7^)	[52]
Nafion/SPAni	30	20	↓ (7.21)	↓ (9.12 × 10^−8^)	[41]
Nafion/PVdF	-	70	↓ (0.59)	↓ (11.7 × 10^−7^)	[54]
PVdF-co-HFP/Nafion	20	20	↑ (31.6)	↑ (1.76 × 10^−6^)	[55]
SPVdF-co-HFP/PBI-coated Nafion	-	-	↓ (15.1)	↓ (4.92 × 10^−7^)	[56]
Nafion/PVA-fiber	5	70	↓ (14)	↓ (3.47 × 10^−6^)	[60]
	10		↓ (11)	↓ (2.83 × 10^−6^)	
Nafion/poly (vinyl alcohol) blend	5	70	↓ (9)	↓ (4.11 × 10^−6^)	[60]
	10		↓ (4.8)	↓ (3.22 × 10^−6^)	
CBA/Nafion-PVA	-	80	↓ (90)	↓ (6.79 × 10^−7^)	[64]
SF-Nafion	-	Room temperature	↑ (130)	↓	[65]
NH-Nafion	-	80	↑ (247)	↓ (4.75 × 10^−7^)	[66]
BFPS-Nafion	-	80	↓ (310)	↓	[67]
Nafion-PPy	-	-	↓ (49.4)	↓ (2.38 × 10^−8^)	[69]
Nafion/CNC	1.5	50, 60, 70	↓	↓	[70]

↑ = higher with respect to Nafion in the respective study; ↓ = lower with respect to Nafion in the respective study; - = not available.

**Table 2:** Summary of the performance of inorganic material modified Nafion membrane based on proton conductivity and methanol permeability.

Modified Nafion Membrane	Filler Content (wt%)	Test Condition Temperature (°C)	Proton Conductivity (mS/cm)	Methanol Permeability (cm^2^/s)	Reference
Nafion-CaO-ZrOH	-	-	↑ (510)	↓ (0.08 × 10^−6^)	[74]
Nafion/ZrP	2.5	60	↑ (41)	↓ (0)	[76]
Nafion/ZrP	2.5	25, 50, 60, 70, 80	↑	-	[75]
Nafion/S-ZrO_2_(NH_3_SO_4_)	30	20	↓ (7.21)	↓ (1.5 × 10^−7^)	[79]
Nafion/S-ZrO_2_	5	25	↓ (78.9)	↓ (0)	[80]
Nafion/S-GO-MOR	5	80	↑ (86.45)	↓	[84]
NH_4_-X/Nafion	5	20, 40, 60, 80	↑	↓	[85]
Nafion/SiO_2_	2.5	30	↓ (115)	-	[86]
Nafion/TiO_2_	2.5	30	↓ (130)	-	[86]
Nafion/Pd-SiO_2_	3	25	↑ (129.2)	↓ (8.36 × 10^−7^)	[89]
SiO_2_@sPS + Nafion	1	25	↑	↓ (2.31 × 10^−8^)	[91]
PVdF/Nafion/SiO_2_–NH_2_	-	80	↑ (210)	↓ (5.2 × 10^−7^)	[93]
Nafion_TiO_2_-RSO_3_H	10	140	↑ (80)	↓ (0.75 × 10^−7^)	[94]
Nafion/CsPW/MMT	-		↑ (3.71)	↓ (1.651 × 10^−6^)	[104]

↑ = higher with respect to Nafion in the respective study; ↓ = lower with respect to Nafion in the respective study; - = not available.

**Table 3:** Summary of the performance of ionic liquids [112].

Ionic Liquid in Nafion-Based Membrane	Methanol Crossover (cm^2^/s)
Tetramethylammonium chloride, TMA^+^ Cl^−^	4.21 × 10^−8^
Phenyltrimethylammonium chloride, TMPA^+^ Cl^−^	5.16 × 10^−8^
n-Dodecyltrimethylammonium chloride, DTA^+^ Cl^−^	3.89 × 10^−8^
Hexadecyltrimethylammonium bromide, CTA^+^ Br^−^	2.59 × 10^−8^
1-Butyl-3-methylimidazolium bis(trifluoromethanesulfonimide), BMIM^+^ Tf_2_N^−^	1.56 × 10^−8^
1-Octyl-3-methylimidazolium bis(trifluoromethanesulfonimide), OMIM^+^ Tf_2_N^−^	1.21 × 10^−8^
Methyl-tricaprylylammonium dicyanamide, ALIQUAT^+^ DCA^−^	4.05 × 10^−9^

**Table 4:** Performance of other Nafion-based membranes according to the major type of filler used.

Filler Type	Membrane	Filler Content	Performance	Reference
Inorganic material	Nafion/sulfonated γ-Fe_2_O_3_	≤1.0 wt%	Induced alignment of sulfonated γ-Fe_2_O_3_, reduced path length for proton movement Increased SO_3_^−^ groups per unit volume, increased IEC Increased water uptake Increased proton conductivity Free volume decreased, retard transportation of methanol molecules 61.4% higher power output than Nafion 117 at 70 °C	[137]
	MoS_2_/Nafion composite membrane MoS_2_+Nafion blending membrane	≤0.5 wt%	MoS_2_/Nafion: better connectivity; better proton conductivity; lower methanol permeability; higher selectivity Strong interaction between (NH_4_)_2_MoS_4_ and Nafion sulfonic acid group MoS_2_+Nafion: poor connectivity; lower proton conductivity; higher methanol permeability; lower selectivity	[138]
	Nafion–h-BN	0.75 wt%	Two times higher water uptake than that of the pristine Nafion membrane Exhibited 58% higher proton conductivity (214 mS/cm) than pristine Nafion (135 mS/cm). 2.5 times higher DMFC peak power density (165 mW/cm^2^) than the pristine Nafion membrane (65 mW/cm^2^)	[139]
Organic material	Sodium dodecyl sulfate/Palladium (SDS/Pd)-modified Nafion	-	Higher proton conductivity in SDS/Pd-Nafion (1.18 × 10^−2^ s/cm) than bare Nafion (0.97 × 10^−2^ s/cm) Yielded higher maximum power density (68.2 mW/cm^2^) than Nafion (62.7 mW/cm^2^) at 2 M methanol and 70 °C	[140]
Metal	Palladium/Nafion	≤3 wt%	Proton conductivity compared to Nafion: -Pd(acac)_2_/Nafion: lower -Pd(thd)_2_/Nafion: higher -Pd(hfa)_2_/Nafion: higher As Pd content was lower, the particles were small and dispersive, so Pd can act as proton conductors; if Pd particles were larger, they might act as barrier Decreased methanol permeability Increased selectivity, better DMFC performance	[141]
Solid acid	CsPW-Nafion	≤10 wt%	Depressed methanol permeation to 7.53 × 10^−8^ cm^2^/s as CsPW content increased to 10 wt% Increased proton conductivity from 3.95 × 10^−2^ mS/cm to 7.25 × 10^−2^ mS/cm at 10 wt% CsPW content due to the water-holding structure Maximum of 101.6% increase in power density relative to recast Nafion	[142]
	PWA and recast Nafion	-	Showed lower methanol permeability (3.59 × 10^−8^ cm^2^/s) than Nafion 115 (104 × 10^−8^ cm^2^/s) Reached higher proton conductivity (58.6 mS/cm) than Nafion 115 (52.9 mS/cm) 20 times higher selectivity than Nafion 115	[143]
Polymer	Nafion/PVFP-BI	-	Reduction in the proton conductivity due to interaction of PBI imidazole C=N- with Nafion −SO_3_H reduced free sulfonic acid groups Decreased methanol crossover due to lower affinity of PVFP-BI toward methanol 5–10 wt% of PBI in PVFP-BI enhanced DMFC performance	[144]
	CHI/PVS-Nafion	-	Reduced methanol permeability by 3–4 folds of that of pristine Nafion Decreased proton conductivity because of the bilayers that blocked the charge carrier Increased water uptake due to incorporation of hydrophilic CHI and PVS	[46]
	BC/Nafion	B: N = 1: 7	Reduced methanol permeability Annealed membrane exhibited less proton conductivity and water uptake than unannealed membrane due to low mobility of protons Improved mechanical properties for annealed membrane Increased power density to a maximum of 20.4 mW/cm	[145]
Metal organic framework	Sulfonated pillar [5] arene/Nafion	≤10 wt%	41% higher of proton conductivity (0.145 S/cm) than Nafion (0.103 s/cm) As content of pillar [5] arene increases, methanol permeability was decreased then increased again due to embedment of some pillar [5] arene molecules on the backbones Lower methanol permeability (2.43 × 10^−6^ cm^2^/s)	[146]
	Nafion-SCONs	≤0.6 wt%	Exhibited high proton conductivity (0.265 S/cm) at 80 °C Decreased methanol permeability to 0.83 × 10^−6^ cm^2^/s, one order magnitude lower than Nafion 44% higher in power density	[147]
	Amino-MIL-53(Al)-Nanosheets@Nafion (AMA@Nafion)	≤2.0 wt%	Higher water uptake (35.1%) than Nafion (27.6%) due to hydrophilic groups (hydroxyl and amino groups) in AMA Decreased proton conductivity Decreased methanol permeability due to reduction in pore size Reached higher maximum power density (23.33 mW/cm) than recast Nafion (20.49 mW/cm)	[148]
Carbon nanomaterials	Nafion/MWCNT-MNP-Nafion	≤0.1 wt%	Critically reduced methanol permeability 2.5-fold increase in proton conductivity, compared to recast Nafion; 50% higher proton conductivity than Nafion 117 5.6-fold increase in power output, compared to recast Nafion; 28% higher power output than Nafion 117 Magnetically driven alignment MWCNT-MNP-Nafion in Nafion resulted in 15-fold increase in selectivity of recast Nafion; 8.7-fold increase in maximum power density	[14]
	Nafion/GO@PDASA	20 layers	93% decrease in methanol permeability due to the decreased in interlayer spacing, which blocked the methanol Maintain the proton conductivity 12.9 times higher selectivity than Nafion	[149]

γ-Fe_2_O_3_—iron oxide; MoS_2_—molybdenum disulfide; h-BN—hexagonal boron nitride; CHI—chitosan; PVS—polyvinyl sulfuric acid; BC—bacterial cellulose; SCONs—sulfonated covalent organic nanosheets; PVFP-BI—poly (vinylidene fluoride-co-hexafluoropropylene) (PVdF-co-HFP) and polybenzimidazole (PBI) blend electrospun nanofiber; MNP—magnetic nanoparticles; PDASA—1,4-phenylenediamine-2-sulfonic acid.

## Data Availability

Not applicable.
